# Enhancing cellulase biosynthesis of *Bacillus subtilis* Z2 by regulating intracellular NADH level

**DOI:** 10.1016/j.isci.2025.112341

**Published:** 2025-04-03

**Authors:** Shuai Liu, Yi Li, Lin Quan, Hai-Xia Liu, Yang Luo, Yong-Zhong Wang

**Affiliations:** 1 Key Laboratory of Biorheological Science and Technology (Chongqing University), Ministry of Education, College of Bioengineering, Chongqing University, Chongqing 400030, China; 2 Chongqing Key Laboratory of Translational Research for Cancer Metastasis and Individualized Treatment, Chongqing University Cancer Hospital, Chongqing 400030, China; 3 Center of Smart Laboratory and Molecular Medicine, NHC Key Laboratory of Birth Defects and Reproductive Health, Jiangjin Hospital, School of Medicine, Chongqing University, Chongqing 400030, China

**Keywords:** Natural sciences, Biological sciences, Biotechnology, Microbial biotechnology, Enzyme engineering

## Abstract

Optimizing cellulase biosynthesis in *Bacillus subtilis* is crucial for enhancing enzymatic yield in lignocellulosic biomass conversion. However, the regulatory mechanisms linking intracellular NAD(H/^+^) levels to cellulase production remain elusive. In this study, we systematically screened 13 genes associated with NAD^+^ biosynthesis and NADH regeneration in *B. subtilis* Z2. Employing a modular engineering strategy with four distinct modules, we directed metabolic flux to enhance NAD^+^ biosynthesis and NADH regeneration. Key genes (*ycel*, *nadV*, *nadM*, *mdh*, and *sucB*) were identified, and their co-expression in *B. subtilis* Z2 resulted in a 13.09-fold increase in intracellular NADH levels and a consequential 2.24- and 2.04-fold enhancement in the filter paper-hydrolyzing (FPase [representing total cellulase]) activity and carboxymethylcellulose (CMCase [representing endoglucanase]) activity, respectively. Experimental validations, including antagonist LaCl_3_ treatment and *spcF* gene deletion, unequivocally established the calcium signaling pathway’s role in regulating cellulase gene overexpression in response to elevated intracellular NAD(H/^+^) levels.

## Highlights

• A modular engineering strategy was used to regulate NAD^+^/NADH level in *B. subtilis*
• The maximal cellulase activity was obtained as assembling *ycel*, *nadV*, *nadM*, and *mdh*
• Cytoplasmic NADH level modified expression of cellulase genes via the Ca^2+^ signaling
• A mechanism on regulation of cellulase synthesis in *B*. *subtilis* was proposed

## Graphical abstract

**Figure:**
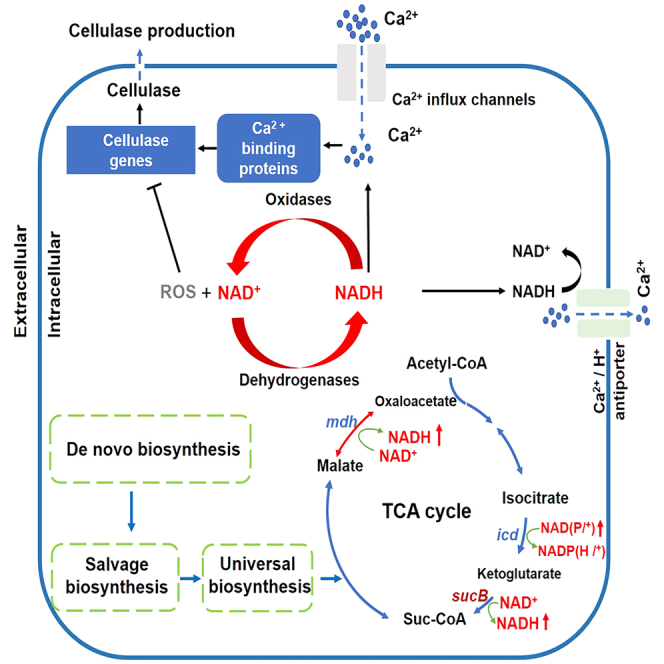

## Introduction

Lignocellulosic biomass, an abundant and sustainable resource, holds significant promise for biofuel and chemical production.^1^ However, the enzymatic hydrolysis of lignocellulose, a pivotal step for industrial applications, poses a bottleneck due to the considerable cellulase consumption.^2^ Urgently needed is the cost-effective production of cellulases. *Bacillus subtilis* presents potential as a cellulase producer.^3^ To improve the cellulase production of *B. subtilis*, researchers have conducted many studies. Mushtaq et al. (2024) utilized potato peel waste as a carbon source and further optimized production conditions using Box-Behnken design based on response surface methodology, resulting in an increase in cellulase production of *B. subtilis* QY4.^4^ Xu et al. (2017) isolated a novel cellulase-producing strain identified as *Bacillus* sp. BS-5. The production of cellulase can be increased by using lactose as the only carbon source.^5^ In addition, our previous study showed that an increase in cellulase production can be enhanced by graphene oxide (GO) stress.^6^ Although enzyme production can be improved through optimization of enzyme production conditions or external stimuli, its high cost of hydrolyzing lignocellulosic biomass into fermentable sugars has a negative impact on the economic benefits of bio-based fuels and chemicals. The imperative to achieve a high cellulase production capacity necessitates a comprehensive elucidation on the regulatory mechanisms governing cellulase biosynthesis in *B. subtilis*. This is critical for the strategic enhancement of cellulase biosynthesis, enabling efficient utilization of lignocellulosic biomass in various industrial applications.

It has been known that the synthesis of cellulase in bacteria is mainly regulated at the transcriptional level, which is also influenced by intracellular signal pathways. So far, several kinds of signaling molecules (*e*.*g*., cAMP and Ca^2+^) have been found to be involved in the expression regulation of cellulase genes in many microbes.^7^^,^^8^ Chen et al. found that the cellulase production could be regulated by cytosolic Ca^2+^ level in *Trichoderma reesei*, an increase in cytosolic Ca^2+^ levels could activate Crz1 (calcineurin-responsive zinc finger 1), and this caused the expression upregulation of the cellulase gene *cbh1* in *T. reesei* as subjected to *N,N*-dimethylformamide induction.^9^^,^^10^ Thus, cellulase biosynthesis was visibly improved.

Nicotinamide adenine dinucleotide (NAD^+^) and its reductant NADH have been known as the key co-factors for intracellular bio-oxidation which plays an important role in cellular functions and energy metabolism, including regulating gene expression and intracellular calcium homeostasis.^11^ Some evidence suggested that NAD(H/^+^) can mediate calcium homeostasis.^12^ NAD(H/^+^) may affect calcium homeostasis through the following pathways: First, NAD^+^ can be decomposed into cyclic adenosine diphosphate ribose (cADPR) through ADP-ribosyl cyclase; cADPR is an endogenous agonist of Ca^2+^ channels effectively mediated by ryanodine receptors.^13^ Besides, Ca^2+^ influx can be regulated by NAD^+^ through promoting P2X_7_R (purinergic receptor) opening to maintain the calcium homeostasis.^14^ Wang et al. (2020) found that the elevated NAD^+^ synthesis could raise the intracellular Ca^2+^ concentration in *Ganoderma lucidum*.^15^^,^^16^ Therefore, it is conceivable that manipulating intracellular NAD(H/^+^) level may lead to cytosolic Ca^2+^ burst in *B. subtilis*, resulting in promotion of cellulase biosynthesis.

Currently, a modular synthetic biology approach has been widely adopted to reconstitute metabolic pathways of cells for improving the objective productivity. Li et al. (2021) found that NAD^+^ in *B*. *subtilis* 168 was constantly generated after transforming the constructed NADH oxidase co-expression plasmid, enhancing xylulose production.^17^ Zhao et al. (2013) confirmed that intracellular NADH supply and carotenoid production were visibly improved through transforming the engineered TCA cycle modular involved in overexpression of malate dehydrogenase or succinate dehydrogenase in *Escherichia coli*.^18^ Furthermore, the construction and optimization of β-ionone biosynthesis pathway by modular engineering strategy increased the yield of β-ionone in *Yarrowia lipolytica*.^19^ However, there are few reports on the use of modular engineering strategies to improve cellulase production in *B*. *subtilis*.

This study pioneers an innovative modular synthetic biology paradigm for precise control of the endogenous NAD(H/^+^) milieu in *B. subtilis*, with the explicit goal of improving cellulase production. Drawing on sophisticated genomic and bioinformatic analyses, a curated selection of 13 genes being intrinsic to the NAD^+^ biosynthesis pathway and NADH regeneration was strategically organized into four distinct engineering modules. Rigorous exploration of module expressions, coupled with meticulous evaluations of their impact on cellulase biosynthesis in *B. subtilis*, unveiled pivotal genes governing intracellular NAD(H/^+^) levels and cellulase production. Moreover, this investigation can unravel the interplay among cytosolic Ca^2+^ concentration, intracellular NAD(H/^+^) level, and the expression level of cellulase genes in *B. subtilis*. The metabolic engineering strategies in this study delineated not only our scientific understanding of the regulatory framework dictating gene expressions in cellulase biosynthesis but also set the stage for approaches to dynamically respond to heightened intracellular NAD(H/^+^) levels in *B. subtilis*.

## Results

### Modular design strategy for increasing intracellular NAD(H/^+^) level

As is well known, NAD^+^ is synthesized through three pathways in *B. subtilis*, i.e., the *de novo* biosynthesis pathway, the salvage biosynthesis pathway, and the universal biosynthesis pathway.^20^^,^^21^

**Figure 1:**
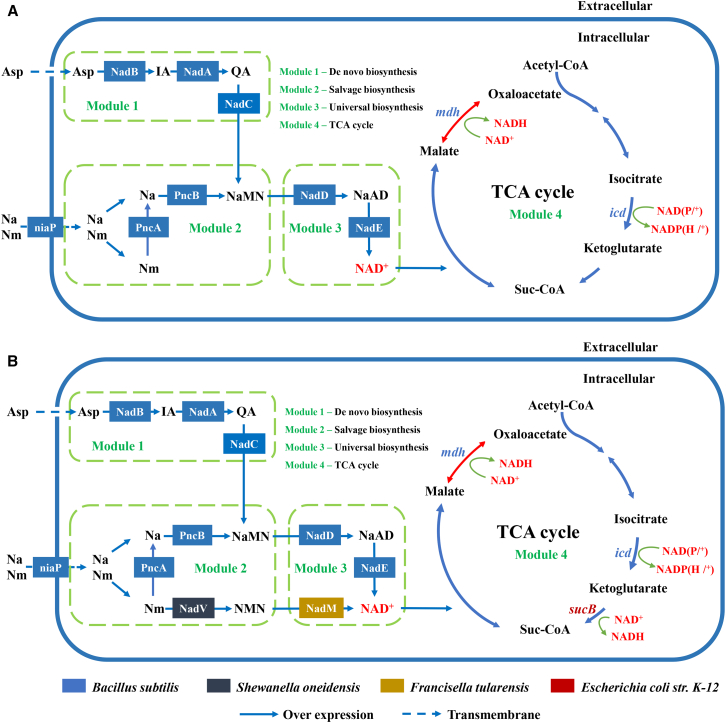
Schematic of modular design to enhance the level of intracellular NAD(H/^+^) in *B. subtilis* Z2 (A) The NAD^+^ biosynthesis and NADH regeneration pathway of wild-type *B. subtilis* Z2 are categorized into four metabolic modules. Module 1–3 is the NAD^+^ synthesis pathway. L-aspartate (L-Asp) is the *de novo* precursor of NAD^+^ in Module 1 (*de novo* pathway). Module 2 involves salvage pathway by assimilating nicotinic acid (Na) and nicotinamide (Nm). Module 3 is the universal biosynthesis pathway as a common part of the *de novo* and salvage pathways. Module 4 (TCA cycle) is NADH regeneration pathway. Genes related to the Module 4 were *mdh*, *icd*, and *sucB*. (B) Recombinant *B. subtilis* Z2 harboring five homogeneously and heterogeneously introduced genes *ycel*, *nadV*, *nadM*, *mdh*, and *sucB* to enhance the level of intracellular NAD(H/^+^) and NADH regeneration. Abbreviations: Asp, aspartate; NMN, nicotinamide mononucleotide; NaMN, nicotinic acid mononucleotide; NaAD, nicotinic acid adenine dinucleotide; TCA, tricarboxylic acid cycle; IA, α-iminosuccinate; Qa, quinolinic acid.

In this work, a modular synthetic biology method for increasing intracellular NAD(H/^+^) level in *B. subtilis* cells was adopted to reveal the intrinsic relationship between total intracellular NAD(H/^+^) level and cellulase biosynthesis (Figure 1A). First, the endogenous genes *nadB, nadA*, and *nadC* in the *de novo* pathway (Modular 1) were engineered for enhancing L-aspartate (L-Asp) assimilation and NAD^+^ synthesis. Besides, *B. subtilis* can assimilate nicotinic acid (Na) and nicotinamide (Nm) as NAD^+^ precursors by niaP transporter (encoded by gene *ycel*). The constructed salvage pathway (Module 2) could convert Nm to nicotinic acid mononucleotide (NaMN) via PncA and PncB in *B. subtilis*. However, *B. subtilis* is incapable of directly converting Nm into NAD^+^ due to lack of the genes *nadV* and *nadM*.^22^^,^^23^ Therefore, the exogenous genes *nadV* (from *Shewanella oneidensis*) in Module 2 and *nadM* (from *Francisella tularensis*) in Module 3 were individually constructed to synergistically drive the metabolic flux from the two precursors toward NAD^+^ synthesis (Figure 1B). The endogenous genes *nadD* and *nadE* in Module 3 were overexpressed to enhance the conversion of NaMN to NAD^+^. To further increase the metabolic flux toward NADH regeneration, two endogenous genes, *mdh* and *icd*, and one exogenous gene *sucB* (from *E*. *coli* K-12) in TCA cycle (Modular 4) were systematically constructed into *B. subtilis* (Figure 1B). The roles and sources of the overexpressed genes (Module 1–4) used in this study are shown in Table 1.

**Table 1:** The roles and sources of the overexpressed genes in this study

Module	Gene	Functional role	E.C.	Molecular weight (kDa)	Source	References
*De novo* biosynthesis (Module 1)	*nadB*	L-aspartate oxidase	1.4.3.16	58.24	*B. subtilis*	Sun et al.^24^
	*nadA*	Quinolinate synthetase	2.5.1.72	41.49	*B. subtilis*	Belda et al.^25^
	*nadC*	Nicotinate-nucleotide pyrophosphorylase	2.4.2.19	31.39	*B. subtilis*	Belda et al.^25^
Salvage biosynthesis (Module 2)	*ycel*	Nicotinic acid and nicotinamide niaP transporter	_	43.7	*B. subtilis*	Rodionov et al.^26^
	*pncA*	Nicotinamidase	3.5.1.19	20.52	*B. subtilis*	Shang et al.^22^
	*pncB*	Nicotinate phosphoribosyl-transferase	6.3.4.21	56.18	*B. subtilis*	Barbe et al.^27^
	*nadV*	Nicotinamide phosphoribosyl-transferase	2.4.2.12	55.1	*S. oneidensis*	Galeazzi et al.^28^
Universal biosynthesis (Module 3)	*nadD*	Nicotinate-nucleotide adenylyltransferase	2.7.7.18	22.16	*B. subtilis*	Barbe et al.^27^
	*nadE*	Ammonium-dependent NAD^+^ synthetase	6.3.1.5	30.40	*B. subtilis*	Eichenberger et al.^29^
	*nadM*	Nicotinamide-nucleotide adenylyltransferase	2.7.7.18	40.4	*F. tularensis*	Rand et al.^30^
TCA cycle (Module 4)	*mdh*	Malate dehydrogenase	1.1.1.37	33.64	*B. subtilis*	Belda et al.^25^
	*icd*	Isocitrate dehydrogenase	1.1.1.42	46.42	*B. subtilis*	Barbe et al.^27^
	*sucB*	Dihydrolipoyl transsuccinylase	2.3.1.61	44.01	*E*. *coli* K-12	Rand et al.^30^

### Optimization of the NAD^+^ biosynthesis modules

#### The *de novo* biosynthesis pathway (Module 1)

**Figure 2:**
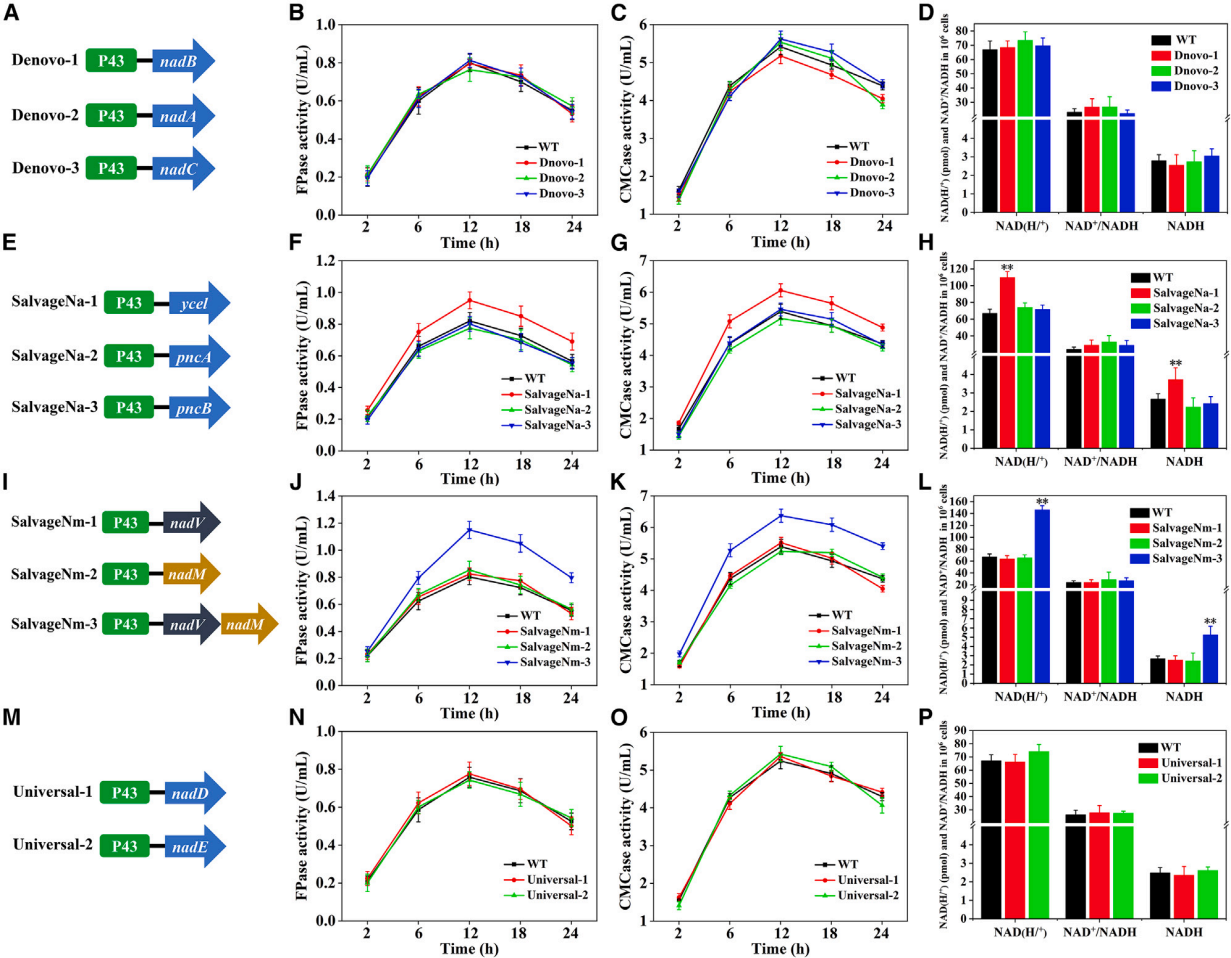
Optimization of the *de novo* pathway (Module 1), salvage pathway (Module 2) and universal pathway (Module 3) to enhance the intracellular NAD^+^ levels and cellulase activity in *B. subtilis* Z2 (A) The construction of three plasmids contained gene *nadB*, *nadA*, and *nadC* in Module 1. (B) FPase activities of the recombinant strains in Module 1. (C) CMCase activities of the recombinant strains in Module 1. (D) Quantitative measurements of intracellular NAD(H/^+^) levels and NAD^+^/NADH ratio of the recombinant strains Denovo-1, -2 and -3 at 12th h of the cultivation. (E) The construction of three plasmids contained the genes *ycel*, *pncA*, and *pncB* in Module 2. (F) FPase activities of the recombinant strains in Module 2. (G) CMCase activities of the recombinant strains in Module 2. (H) Quantitative measurements of intracellular NAD(H/^+^) levels and NAD^+^/NADH ratio of the recombinant strains SalvageNa-1, -2 and -3 at 12th h of the cultivation. (I) The construction of three plasmids contained the genes *nadV* in Module 2 and *nadM* in Module 3 and assemblage of the genes (*nadV* and *nadM*) in the Nm-utilization pathway of Module 2 and Module 3. (J) FPase activities of the recombinant strains in Module 2 and Module 3. (K) CMCase activities of the recombinant strains in Module 2 and Module 3. (L) Quantitative measurements of intracellular NAD(H/^+^) levels and NAD^+^/NADH ratio of the recombinant strains SalvageNm-1, -2 and -3 at 12th h of the cultivation. (M) The construction of two plasmids contained the genes *nadD* and *nadE* in Module 3. (N) FPase activities of the recombinant strains in Module 3. (O) CMCase activities of the recombinant strains in Module 3. (P) Quantitative measurements of intracellular NAD(H/^+^) levels and NAD^+^/NADH ratio of the recombinant strains Universal-1 and -2 at 12th h of the cultivation. Values are the mean ± SD of the results from three independent experiment. Asterisks indicate significant differences (∗*p* < 0.05, ∗∗*p* < 0.01, Student’s *t* test).

Increasing precursor supply is a common strategy to increase metabolic flux toward desired products.^31^ Here, to improve Asp conversion to NAD^+^, three endogenous genes (*nadB*, *nadA*, and *nadC*) in the *de novo* pathway (Module 1) were individually overexpressed in *B. subtilis* by constructing three recombinant strains, namely Denovo-1, Denovo-2 and Denovo-3 (Figure 2A). The results of SDS-PAGE and qPCR show that these genes involved in the *de novo* biosynthesis pathway were overexpressed at both translational and transcriptional levels (Figures S4A and S5A). However, it brought a low impact on the activities of FPase and CMCase (Figures 2B and 2C), biomass (Figure S6B), FPase activity/biomass (Figure S7A), CMCase activity/biomass (Figure S8A), intracellular NAD(H/^+^) level and NAD^+^/NADH ratio in the recombinant strains, as compared to WT (Figure 2D). This reveals that cellulase activity and NAD^+^ biosynthesis in *B. subtilis* was insensitive to the overexpression of these *de novo* pathway genes.

#### The salvage biosynthesis pathway (Module 2)

In the salvage pathway, NAD^+^ is generated directly from the vitamin precursors, including Na and Nm.^32^ Previous studies demonstrate that niaP is a bifunctional transporter for transferring Na and Nm in *B. subtilis*.^26^^,^^33^ PncA can catalyze the conversion of Nm to Na by deamination, and then Na is converted to NaMN by PncB.^22^ Therefore, to improve the utilization of Na and Nm, the three endogenous genes (*ycel*, *pncA*, and *pncB*) were individually overexpressed in *B. subtilis*, namely Salvage Na-1, -2, and -3 (Figure 2E). Notably, Salvage Na-1 reached the maximal FPase and CMCase activities of 0.95 ± 0.02 U/mL and 6.06 ± 0.36 U/mL, which were 1.16- and 1.13-fold that of WT (0.82 ± 0.03 U/mL and 5.38 ± 0.29 U/mL), respectively (Figures 2F and 2G). The maximal biomass (1.74 ± 0.32 × 10^8^ CFU/mL) was also achieved after incubation of 18 h in Salvage Na-1, which was 1.12-fold that of the control group (1.55 ± 0.28 × 10^8^ CFU/mL) (Figure S6C). In contrast, there was a negligible difference in cellulase activity and biomass between WT and Salvage Na-2, and-3 (Figures 2F, 2G, and S6C). Figures S7B and S8B showed that the cellulase activity/biomass of Salvage Na-1 was higher than that of other strains, and reached the highest value in the 6^th^ h. This result demonstrates that the overexpression of gene *ycel* could improve cell growth and cellulase activity. To determine whether the improvement of cellulase activity originated from the change in electron pool, the intracellular NAD(H/^+^) and NADH levels and NAD^+^/NADH ratios of Salvage Na-1, -2, -3 and WT were measured, respectively. As shown in Figure 2H, the intracellular NAD(H/^+^) and NADH levels of Salvage Na-1 were 1.64- and 1.39-fold that of WT. The translational and transcriptional levels of the gene *ycel* were consistent with the levels of intracellular NAD(H/^+^) and NADH (Figures S4B and S5B). Besides, the NAD^+^/NADH ratio of Salvage Na-1 almost unchanged, as compared with WT (Figure 2H), suggesting that the gene expression didn’t cause obvious effect on the intracellular redox state.

#### The shortcut NAD^+^ biosynthesis pathway (Module 2 and 3)

Nicotinamide phosphoribosyl-transferase (encoded by gene *nadV* from *S. oneidensis*) has been identified as an enzyme that catalyzes Nm to NMN.^28^ The latter can be transformed to NAD^+^ by nicotinamide-nucleotide adenylyltransferase (encoded by gene *nadM* from *F. tularensis*).^34^ However, Nm uptake in *B. subtilis* can’t be reinforced due to the lack of the intracellular nadV and nadM in the salvage pathway, as a result, NAD^+^ biosynthesis can’t be promoted. In this work, to relieve effect of the rate-limiting steps and shorten the conversion pathways of Nm to NAD^+^, the exogenous gene *nadV* (from *S. oneidensis*) and *nadM* (from *F. tularensis*) were individually overexpressed or were simultaneously reconstructed in *B. subtilis*, obtaining three recombination strains (SalvageNm-1, SalvageNm-2, and SalvageNm-3) (Figure 2I). As shown in Figures 2J, 2K, S6D, S7C, and S8C the activities of FPase and CMCase, biomass, FPase activity/biomass and CMCase activity/biomass of SalvageNm-3 were always significantly higher than those of the control.

As shown in Figures 2J–2L, single overexpression of the genes *nadV* and *nadM* in *B. subtilis* caused a negligible impact on cellulase activities, intracellular NAD(H/^+^) level and NAD^+^/NADH ratio, as compared with WT. SDS-PAGE and qPCR results demonstrate that the genes (*nadV* and *nadM*) were successfully overexpressed (Figures S4B, S4C, S5B, and S5C). On the contrary, the cellulase activities, intracellular levels of NAD(H/^+^) and NADH in SalvageNm-3 (synchronous expression of *nadV* and *nadM*) significantly increased. It appears that the shortcut pathway in *B. subtilis* may have the potential to promote the metabolic flux from Nm to NAD^+^. Therefore, the genes *ycel*, *nadV*, and *nadM* were selected for subsequent modular assembly.

#### The universal biosynthesis pathway (Module 3)

As a common part of the *de novo* and the salvage pathways, the universal pathway converts NaMN to NAD^+^ by NadD and NadE in *B. subtilis*.^35^ To drive the precursors (L-Asp, Na, and Nm) to redirect toward NAD^+^ biosynthesis, the engineering strains (Universal-1 and Universal-2) overexpressed the genes *nadD* and *nadE* were constructed (Figure 2M). SDS-PAGE and qPCR results demonstrate that the genes (*nadD* and *nadE*) in module 3 were successfully overexpressed (Figures S4C and S5C). However, the FPase and CMCase activities (Figures 2N and 2O), biomass (Figure S6E), FPase activity/biomass (Figure S7D), CMCase activity/biomass (Figure S8D) and intracellular levels of NAD(H/^+^) (Figure 2P) in the recombinant strains were almost the same as those in WT, suggesting that the overexpression of those genes caused little effect on NAD^+^ biosynthesis and cellulase activity.

All the above results indicate that the increase in the intracellular NAD(H/^+^) level significantly improved cellulase activity, biomass and cellulase activity/biomass. This implied that the enhancement of cellulase is due to an increase in biomass and enzyme production by individual cells. However, whether the increase in NADH or NAD^+^ levels promote cellulase biosynthesis needs to be further investigated.

### Optimization of the NADH regeneration module

**Figure 3:**
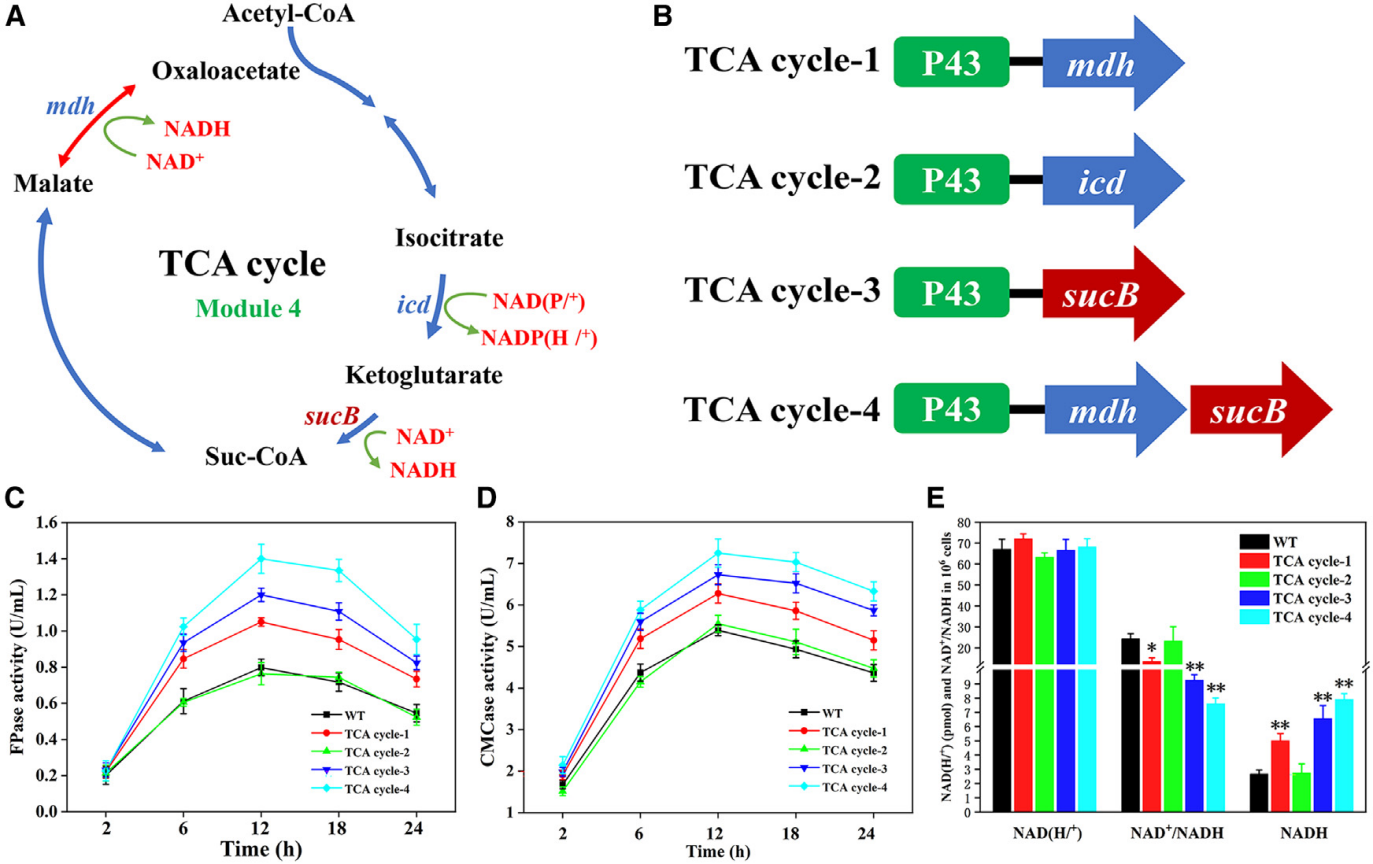
Optimization of TCA cycle (Module 4) in *B. subtilis* (A) A depiction of the overexpressed genes in Module 4. (B) The construction of four plasmids that included genes *mdh*, *icd*, and *sucB* and assemblage of genes (*mdh* and *sucB*) in Module 4. (C) FPase activities of the recombinant strains in Module 4. (D) CMCase activities of the recombinant strains in Module 4. (E) Quantitative measurements of intracellular NAD(H/^+^) levels and NAD^+^/NADH ratios of the recombinant strains (TCA cycle-1, -2, -3, and -4) at 12th h of the inoculation. Values are the mean ± SD of the results from three independent experiment. Asterisks indicate significant differences (∗*p* < 0.05, ∗∗*p* < 0.01, Student’s *t* test).

NADH is not only the main electron donor of the respiratory chain, but also the principal antioxidant in cells.^36^ To confirm effect of intracellular NADH or NAD^+^ levels on cellulase production, the metabolic flux in TCA cycle for NADH regeneration was redirected in *B. subtilis*. Here, the endogenous genes *mdh*, *icd*, and an exogenous gene *sucB* (from *E*. *coli* K-12) were overexpressed individually or synchronously in *B. subtilis*, obtaining four strains (TCA cycle-1, TCA cycle-2, TCA cycle-3, and TCA cycle-4) (Figures 3A and 3B). The SDS-PAGE and qPCR results demonstrate that the expressions of the three genes increased at both the translational and transcriptional levels (Figures S4D and S5D).

As shown in Figure 3C, the maximal FPase activities of TCA cycle-1 (1.05 ± 0.02 U/mL), TCA cycle-3 (1.20 ± 0.01 U/mL), and TCA cycle-4 (1.41 ± 0.02 U/mL) were 1.33-, 1.52- and 1.78-fold that of the WT (0.79 ± 0.03 U/mL) after incubation for 12 h, respectively. Similarly, the maximal CMCase activities of TCA cycle-1 (6.28 ± 0.35 U/mL), TCA cycle-3 (6.73 ± 0.41 U/mL), and TCA cycle-4 (7.25 ± 0.53 U/mL) were 1.16-, 1.24- and 1.34-fold that of the WT (5.41 ± 0.28 U/mL) after incubation for 12 h, respectively (Figure 3D). It indicates that individual or synchronous overexpression of the genes *mdh* and *sucB* could enhance the cellulase activity of *B. subtilis*. The change of their biomass and cellulase activity/biomass was the same as the corresponding enzyme activity (Figures S6F, S7E, and S8E). Furthermore, the levels of NAD(H/^+^) and NADH, as well as NAD^+^/NADH ratio, were also examined. As shown in Figures 3C–3E, the intracellular levels of NADH were significantly increased in TCA cycle-1, -3, and -4, and the cellulase activity was improved with the increase in intracellular NADH levels. Although the total levels of NAD(H/^+^) in the three recombinant strains were similar, the NAD^+^/NADH ratio significantly reduced in TCA cycle-1, TCA cycle-3, and TCA cycle-4 (Figure 3E). It may be ascribed to an increased conversion efficiency of malate to oxaloacetate because of overexpression of the gene *mdh*. Moreover, the overexpression of the gene *sucB* promoted the oxidative decarboxylation of ketoglutarate to form suc-CoA. As a result, NADH regeneration from NAD^+^ was facilitated.^37^^,^^38^ According to above results, it can be inferred that the increase in intracellular NADH level and the decrease in NAD^+^/NADH ratio could stimulate cellulase production in *B. subtilis*. Therefore, the genes *mdh* and *sucB* in TCA cycle (Module 4) were selected for subsequent modular assembly.

### Integration of the regulation modules on the intracellular NADH level and cellulase biosynthesis

**Figure 4:**
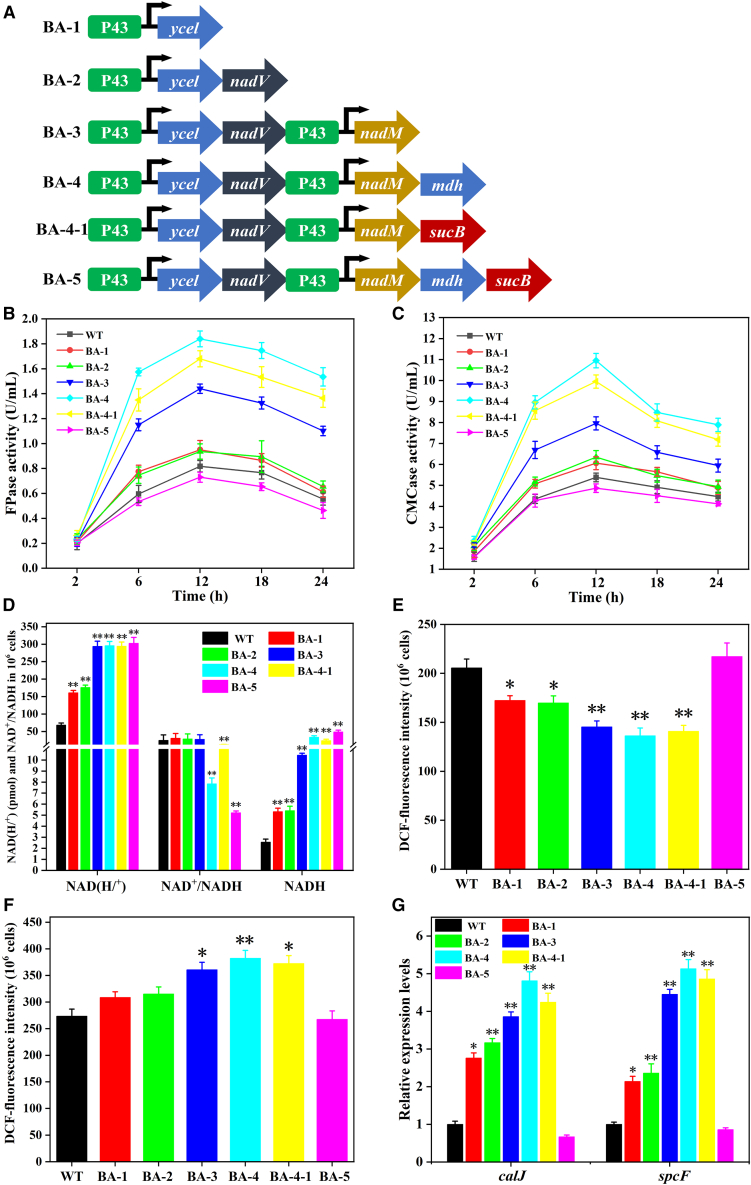
Modular assembly of the crucial genes for regulating intracellular NADH levels and NAD^+^/NADH ratios to enhance the cellulase production (A) The construction of five plasmids that included gene *ycel*, *nadV*, *nadM*, *mdh*, and *sucB* in Module1-4. (B) FPase activities of the WT and the recombinant strains BA-1 to BA-5. (C) CMCase activities of the WT and the recombinant strains BA-1 to BA-5. (D) Quantitative measurement of intracellular NAD(H/^+^) and NADH levels and NAD^+^/NADH ratios of the recombinant strains at 12^th^ h of cultivation. (E) The contents of cytosolic ROS in the recombinant strains. (F) The contents of cytosolic Ca^2+^ in the recombinant strains. (G) The expression levels of Ca^2+^ homeostasis-related genes *calJ* (encoding pH-sensitive calcium-leak permease) and *spcF* (encoding calcium-binding domain protein) in BA-1 to BA-5. Values are the mean ± SD of the results from three independent experiment. Asterisks indicate significant differences (∗*p* < 0.05, ∗∗*p* < 0.01, Student’s *t* test).

To further improve the intracellular level of NADH, the five identified genes (*ycel*, *nadV*, *nadM*, *mdh*, and *sucB*) involved in the above four modules were assembled, respectively, obtaining six engineered strains (BA-1 to BA-5, Figure 4A). SDS-PAGE results demonstrate that the translational levels of the five genes increased in *B. subtilis* (Figures S4E and S4F). As shown in Figures 4B and 4C, the cellulase activities gradually increased with the step-by-step transformation of the four genes *ycel*, *nadV*, *nadM*, and *mdh* or *ycel*, *nadV*, *nadM*, and *sucB*. The maximal FPase activity (1.84 ± 0.04 U/mL) and CMCase activity (10.95 ± 1.78 U/mL) were achieved in BA-4 with the co-expression of four genes (*ycel*, *nadV*, *nadM*, and *mdh*) after incubation for 12 h, which were 2.24- and 2.04-fold than that of WT (0.81 ± 0.02 U/mL and 5.37 ± 0.37 U/mL), respectively. The qPCR results suggest that the transcriptional levels of the four genes were upregulated in BA-4 (Figure S5E). However, the minimal FPase activity (0.73 ± 0.02 U/mL) and CMCase activity (4.68 ± 0.23 U/mL) were found in BA-5 (*ycel*, *nadV*, *nadM*, *mdh*, and *sucB*), which were 0.89- and 0.87-fold of WT, respectively (Figures 4B and 4C).

As shown in Figure S9, biomass and cellulase activity/biomass of the engineered strains were gradually increased with step-by-step integration of the four genes *ycel*, *nadV*, *nadM*, and *mdh* or *ycel*, *nadV*, *nadM*, and *sucB*. The maximal biomass concentration (1.98 ± 0.41 × 10^8^ CFU/mL) was achieved in BA-4 after incubation of 18 h, which was 1.25-fold that of the control group (1.59 ± 0.37 × 10^8^ CFU/mL). In addition, BA-4 has the highest cellulase activity/biomass after incubation of 6 h. However, the biomass and cellulase activity/biomass of BA-5 was almost the same as that of the control group. This means that synchronous overexpression of the four genes *ycel*, *nadV*, *nadM*, and *mdh* could improve cellulase synthesis and cell growth of *B. subtilis*.

To reveal the relationship between the cellulase activity and intracellular electron pool, the intracellular NAD(H/^+^) levels and NAD^+^/NADH ratios of the WT and the five recombinant strains were measured, respectively. As compared with the WT, the levels of intracellular NAD(H/^+^) significantly increased with step-by step integration of the three genes *ycel*, *nadV*, and *nadM* in BA-1 to BA-3, whereas there is a negligible difference in BA-3 to BA-5 (Figure 4D). It demonstrates that the genes *mdh* and *sucB* caused a little influence on the biosynthesis of intracellular NAD(H/^+^). Of note, the levels of intracellular NADH in BA-1 to BA-5 were 2.07-, 2.11-, 4.08-, 13.09-, 9.21- and 19.03-fold that of the WT, respectively (Figure 4D). However, the NAD^+^/NADH ratio of BA-4 and BA-5 were only 7.84 and 5.22, respectively, which reduced by 68.10% and 78.76%, respectively, as compared to the WT (24.58). The result suggests that step-by-step overexpression of the five identified genes (*ycel*, *nadV*, *nadM*, *mdh*, and *sucB*) redistributed the metabolic flux toward the intracellular NADH biosynthesis. Furthermore, the consumption of Na and Nm in these engineered strains were quantified. The results showed that both the Na and Nm consumption was consistent with the increase in NAD(H/^+^) level of the strains, indicating that the metabolic flux from the precursors toward the NAD(H/^+^) biosynthesis (Figure S10). Although the maximal intracellular NADH level was achieved in BA-5 among all the engineered strains, the minimal cellulase activity was observed (Figures 4B and 4C). This may mean that excessive NADH in cells could produce “reductive stress”, which might induce ROS accumulation through oxidizing NADH. Previous studies have shown that excessive accumulation of NADH in cells can lead to xanthine oxidase/xanthine dehydrogenase oxidizing NADH to generate ROS, resulting in intracellular “reducing stress”.^39^ To determine whether the reduction of cellulase activity originated from the “reductive stress” in BA-5, the endogenous ROS contents of the WT and the five recombinant strains were measured, respectively. As shown in Figures 4E and S11, the endogenous ROS contents significantly reduced with the step-by-step integration of the four genes in BA-1 to BA-4-1, respectively. Nevertheless, the level of ROS in BA-5 was visibly higher than that of the WT. This did not only impact the intracellular redox state but also broke the intracellular homeostasis balance (such as calcium homeostasis).^40^ The results seem to suggest that the decrease in cellulase activity of BA-5 could potentially be related to “reductive stress”.

As is well known, NAD^+^ and NADH can mediate intracellular calcium homeostasis.^41^ In bacteria, to maintain cellular Ca^2+^ homeostasis, cells can uptake Ca^2+^ through Ca^2+^ channels or excrete Ca^2+^ through Ca^2+^ transporters driven by electrochemical potential or Ca^2+^-translocating ATPases. *calJ* (ID: 937389) was responsible to synthesis of a Ca^2+^ leakage channel protein YetJ in *B. subtilis*,^42^ which can transport extracellular Ca^2+^ into the cytoplasm. The calmodulin-like protein spcF (encoded by gene *spcF*, ID: 937389) plays an important role in calcium signaling transduction.^43^ As shown in Figure 4F, the cytosolic Ca^2+^ levels from BA-1 to BA-4 increased gradually, whereas the index in BA-5 was lower than that of the WT. This change was consistent with that of cellulase activity (Figures 4B and 4C). Moreover, the transcriptional levels of Ca^2+^ homeostasis-related genes *calJ* and *spcF* in BA-1 to BA-4 were gradually upregulated (Figure 4G). Therefore, it is possible to suggest that the intracellular NADH level may have an impact on cellulase activity through regulating the cytosolic Ca^2+^ level.

### The expression of cellulase genes via calcium signaling

To verify the above deduction, intracellular Ca^2+^ level was detected by Fluo-3 AM fluorescent dye method. LaCl_3_ (a plasma membrane Ca^2+^ channel blocker) was added into the culture medium to prevent the influx of extracellular Ca^2+^. Moreover, to further clarify the role of calcium signaling in the regulation on cellulase biosynthesis, the cellulase activities and the expression levels of cellulase genes in the control groups (WT, BA-3 and BA-4), LaCl_3_ groups (WT + LaCl_3_, BA-3 + LaCl_3_ and BA-4 + LaCl_3_) and Δ*spcF* groups (Δ*spcF*::WT, Δ*spcF*::BA-3 and Δ*spcF*::BA-4 strains) (Table S3) were detected, respectively.

**Figure 5:**
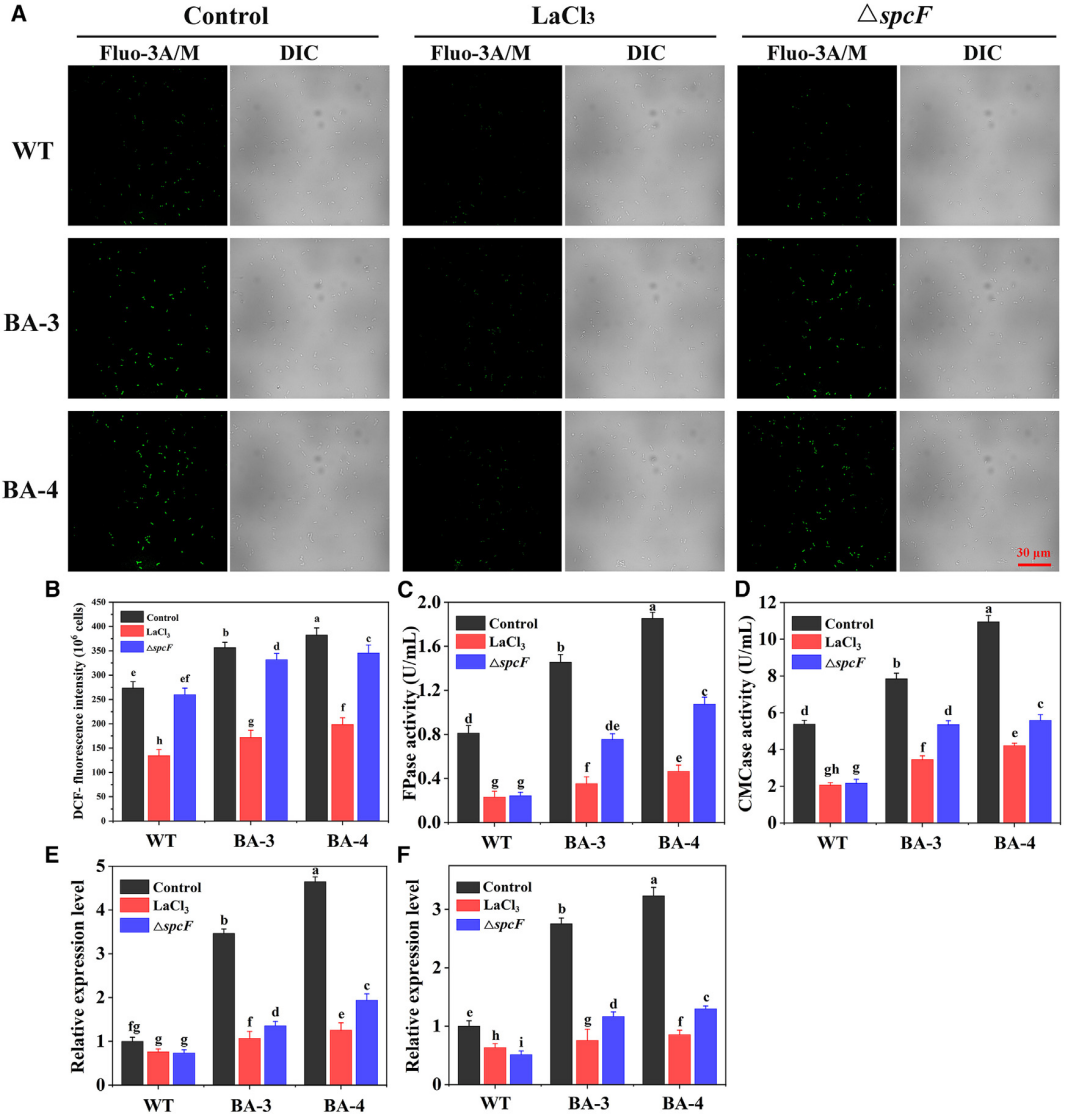
Effects of Ca^2+^ inhibitor and knockout of the gene *spcF* on cytosolic Ca^2+^ contents and cellulase production in WT, BA-3 and BA-4 (A) Effects of Ca^2+^ inhibitor and *spcF* on the fluorescence intensity, Bar, 30 μm, (B) relative fluorescent ratio, (C) FPase activity, (D) CMCase activity, (E) expression levels of *eglS*, (F) expression levels of *bglC*. Values are mean ± standard deviation (*n* = 3). Different letters indicate significant differences between the columns (*p* < 0.05, Duncan’s multiple-range test).

As shown in Figures 5A and 5B, the green fluorescence intensities in LaCl_3_ groups significantly decreased as compared to the control groups, suggesting the cytosolic Ca^2+^ burst was effectively blocked after adding LaCl_3_. Furthermore, the green fluorescence intensities of Δ*spcF*::BA-3 and Δ*spcF*::BA-4 were higher than that of the Δ*spcF*::WT, which further verify that NADH mediated an increase in cytosolic Ca^2+^ level. However, after knocking out the *spcF* gene, the intracellular Ca^2+^ concentration of all strains (WT, BA-3, and BA-4) was almost the same as that of the control group. Therefore, it can be inferred that the accumulation of cytoplasmic Ca^2+^ in *B. subtilis* is almost unaffected by spcF.

The cellulase activities and expression levels of the cellulase-related genes *eglS* (encoding endo-1,4-beta-glucanase) and *bglC* (encoding aryl-phospho-beta-D-glucosidase) were detected in all runs. It is found that FPase and CMCase activities and transcriptional levels of *eglS* and *bglC* in LaCl_3_ groups or Δ*spcF* groups visibly decreased as compared to those in the control groups (Figures 5C and 5D). The transcriptional levels of *eglS* and *bglC* in the LaCl_3_ groups (BA-3 + LaCl_3_ and BA-4 + LaCl_3_) and Δ*spcF* groups (Δ*spcF*::BA-3 and Δ*spcF*::BA-4) were significantly decreased than those of the control groups (BA-3 and BA-4) (Figures 5E and 5F). The result demonstrates that blocking the Ca^2+^ leakage channel or deletion of the gene *spcF* encoding Ca^2+^ binding protein could significantly attenuate on cellulase biosynthesis in *B. subtilis* Z2. It can be concluded that calcium signaling pathway was involved in the overexpression of cellulase-related genes in *B. subtilis* Z2.

## Discussion

Studies have shown that increasing intracellular NAD(H/^+^) level in microbial cells using the module strategy of cell reconstruction could improve the yield of target products.^27^ Li et al. (2021) found that L-xylulose production was enhanced through transforming the constructed co-expression plasmid of NADH oxidase into *B*. *subtilis* due to an increase in intracellular NAD^+^ level.^17^ Zhao et al. (2013) confirmed that overexpression of malate dehydrogenase or succinate dehydrogenase through the engineered TCA cycle module promoted NADH supply and carotenoid production.^18^ Although the regulation of intracellular NAD(H/^+^) on cellular metabolism and other objective production has been engaged, the regulation mechanism of intracellular NAD(H/^+^) level on cellulase-related gene expressions is still unclear.

Currently, three NAD^+^ biosynthesis pathways have been found in *B*. *subtilis*, i.e., the *de novo* pathway, the salvage pathway, and the universal pathway.^20^ The *de novo* pathway is involved in NAD^+^ biosynthesis when the availability of niacin is restricted.^44^ In the *de novo* pathway, NaMN is synthesized from L-Asp through the consecutively catalytic reactions of NadB, NadA, and NadC.^41^ In the salvage pathway, Nm is first converted to Na by PncA, then Na is catalyzed to NaMN by PncB.^22^ As a common portion of *de novo* pathway and salvage pathway, the universal pathway can convert NaMN to NAD^+^ through NadD and NadE. In some species, NAD^+^ is directly synthesized from Nm through a two-step reaction, that is, Nm is first converted to NMN by NadV and the latter is then catalyzed to NAD^+^ by NadM.^25^ In this work, Module 1 (the *de novo* pathway), Module 2 (the salvage pathway), and Module 3 (the universal pathway) were constructed and transformed into *B. subtilis* cells, respectively, which enabled the redirection of the metabolic flux toward NAD^+^ biosynthesis in the engineered strain *B. subtilis* (Figure 1; Table 1). In addition, to shorten the biosynthesis pathway of Nm to NAD^+^, the exogenous gene *nadV* (from *S. oneidensis*) and *nadM* (from *F. tularensis*) were overexpressed individually or synchronously in *B. subtilis* (Figure 2I). As a result, the intracellular NAD(H/^+^) level, cellulase activity, and biomass of *B. subtilis* significantly increased in the engineered strains (Figures 2F–2H, 2J–2L, S6C, and S6D). Besides, as a part of the central carbon metabolism, the TCA cycle is involved in NADH regeneration.^45^^,^^46^ As shown in Figures 3C–3E and S6F, the maximal intracellular NADH level, cellulase activity, and biomass concentration were achieved in TCA cycle-4 (co-overexpression of the genes *mdh* and *sucB*). Thus, the five genes *ycel*, *nadV*, *nadM*, *mdh*, and *sucB* were selected for subsequent modular assembly in *B. subtilis* for improving NAD(H/^+^) biosynthesis and cellulase production.

According to the above results, six engineered *B. subtilis* strains (BA-1 to BA-5, Figure 4A) were successfully assembled, respectively. Among these recombinant strains, the maximal cellulase activity of 10.95 ± 1.78 U/mL was obtained in BA-4 with the co-expression of four genes (*ycel*, *nadV*, *nadM*, and *mdh*) (Figures 4B and 4C). In comparison to other cellulase producing microorganisms, the cellulase activity of BA-4 is higher, indicating that the enzyme yield can be improved more effectively through modular engineering strategy (Table S6). However, the minimal cellulase activity was found in BA-5 with co-expression of the five genes (*ycel*, *nadV*, *nadM*, *mdh*, and *sucB*). The NAD^+^/NADH ratio of BA-5 reduced by 78.76% than that of the WT (Figure 4D). Furthermore, the cytosolic ROS level significantly increased in BA-5 compared with the WT and BA-1 to BA-4 (Figures 4E and S11). This might be ascribed to the fact that the massive accumulation of intracellular NADH could disrupt the physiological homeostasis in BA-5 due to the “reductive stress”. The reductive stress resulted from the activation of xanthine oxidase/xanthine dehydrogenase to generate ROS by oxidizing NADH.^39^

Currently, the effect of intracellular NAD(H/^+^) level on calcium signaling has also attracted increasing attention. NAD^+^ and NADH can promote Ca^2+^ release from endogenous Ca^2+^ stores and regulate Ca^2+^ influx from the extracellular environment.^47^ Chen et al. (2021) have demonstrated that the increased intracellular Ca^2+^ level played an important role in regulating lignocellulolytic enzyme synthesis through the calcineurin-Crz1 signaling cascade in *T. reesei*.^48^ In bacteria, Ca^2+^ binding proteins such as calmodulin-like protein may function as Ca^2+^ sensors which transfer the stimuli to downstream target proteins and regulate gene expression through cascade reactions.^49^ Here, as compared with the control groups, the cellulase activity in the LaCl_3_ and Δ*spcF* groups significantly decreased (Figure 5). Therefore, there is an indication that the increased intracellular NADH level might upregulate the expression of cellulase genes via the calcium signaling in *B. subtilis*.

**Figure 6:**
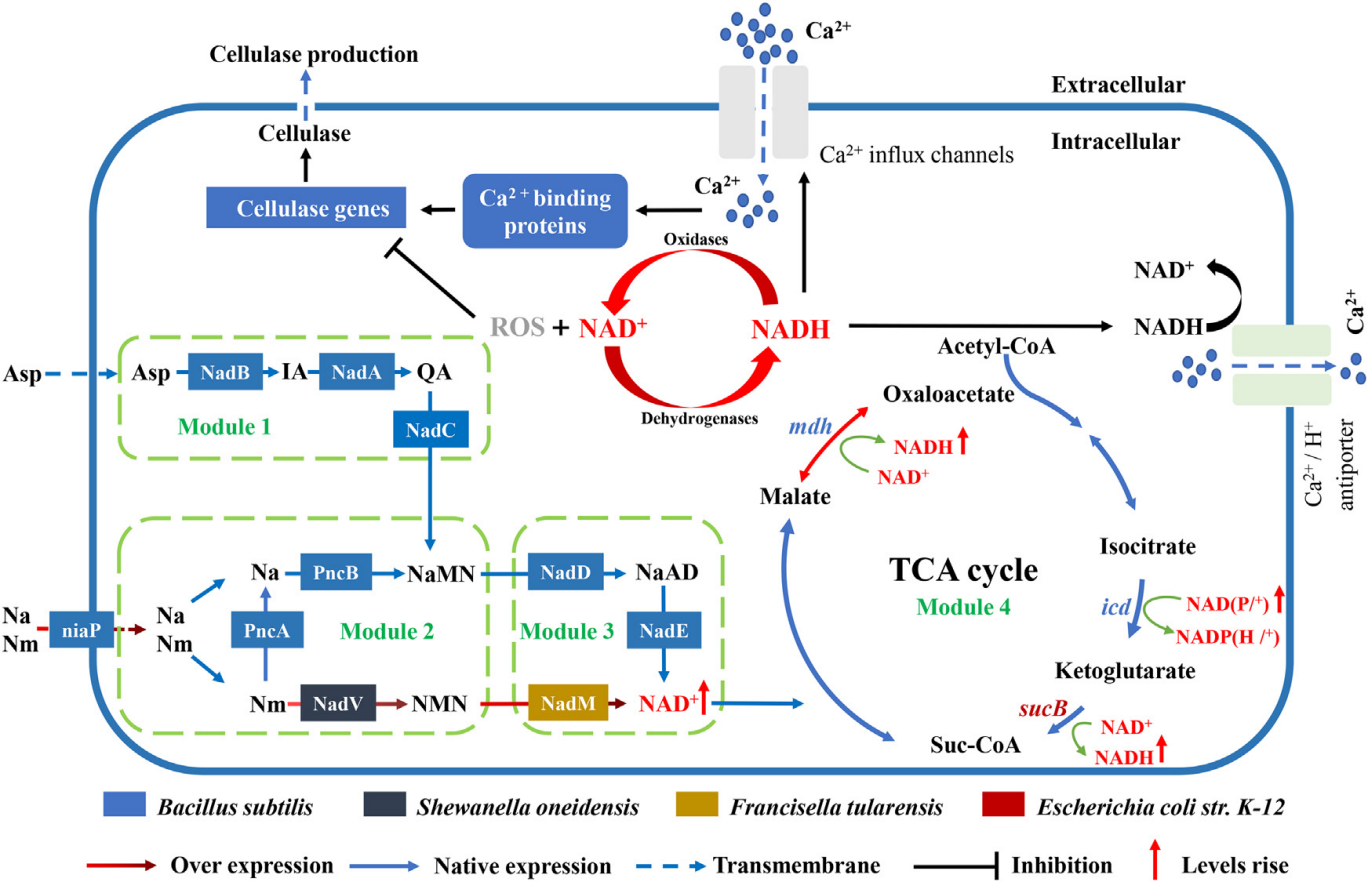
Mechanistic model of the intracellular NAD(H/^+^) level regulating cellulase production in *B. subtilis* Solid arrows indicate data supported by this work; dashed arrows indicate undefined regulation.

The key takeaway in this study is to successfully promote cellulase biosynthesis in *B. subtilis* adopting a modular synthetic biology strategy. By strategically manipulating metabolic flux to increase intracellular NAD(H/^+^) levels, we identified and overexpressed the crucial genes that significantly influenced cellulase activity. Notably, the co-overexpression of *ycel, nadV, nadM*, and *mdh* in the recombinant strain BA-4 resulted in a substantial elevation of the intracellular NADH levels and the maximal cellulase activity. Our findings reveal the modulation mechanism on which the elevated intracellular NADH levels trigger calcium signaling, following the expression of cellulase-related genes, was upregulated, resulting in enhancement of intracellular cellulase biosynthesis and an increase in intracellular cellulase activity (Figure 6). This study not only introduces an approach for improving cellulase production but also provides valuable insights into the intricate regulatory mechanisms governing cellulase biosynthesis in *B. subtilis*, opening avenues for future advancements in biotechnological applications.

### Limitations of the study

In this article, we employed a modular synthetic biology strategy to enhance cellulase biosynthesis in *B. subtilis* by systematically screening and co-expressing key genes to elevate intracellular NADH levels. However, this study still has many limitations, such as: (1) The research focused solely on *B. subtilis* Z2, and the generalizability of the findings to other microbial systems remains unexplored. (2) While the modular engineering strategy successfully enhanced NAD(H/^+^) levels and cellulase activity, the long-term stability and scalability of the engineered strains under industrial conditions were not assessed. (3) The study primarily investigated the calcium signaling pathway’s role in cellulase regulation, leaving other potential regulatory mechanisms unaddressed. (4) The metabolic burden imposed by the overexpression of multiple genes (e.g., *ycel*, *nadV*, *nadM*, and *mdh*) on cellular fitness and growth was not thoroughly evaluated. (5) The environmental and economic feasibility of implementing this strategy in large-scale industrial applications requires further investigation. These limitations highlight the need for future studies to address these gaps and optimize the approach for broader applicability.

## Resource availability

### Lead contact

Further information and requests for resources and reagents should be directed to and will be fulfilled by the lead contact, Dr. Yong-Zhong Wang (wangyzh@cqu.edu.cn).

### Materials availability

This study did not generate new unique reagents.

### Data and code availability

• Strain information has been deposited at NCBI database. They are publicly available as of the date of publication. Accession numbers are listed in the key resources table. RT-qPCR data reported in this paper will be shared by the lead contact upon reasonable request.
• All data can be obtained from the lead contact, provided the request is reasonable.
• This paper does not report original code.

## Acknowledgments

The research work was financially supported by the Key National Research and Development Program of China (2022YFC2106203, 2022YFC2009603), the 10.13039/501100001809National Natural Science Foundation of China (82372355, 82241059), the Yunnan Province Major Science and Technology Special Project (202302AA310039).

## Author contributions

**S.L.**: Methodology, experiments, data analysis, and writing draft. **Yi Li**: Methodology, experiments, and data analysis. **L.Q.**: Methodology and experiments. **H.-X.L.**: Conceptualization and writing–reviewing and editing. **Yang Luo**: Conceptualization and methodology. **Y.-Z.W.**: Fund supporting and supervising all this work.

## Declaration of interests

The authors declare no competing interests.

## STAR★Methods

### Key resources table

**Table:** 

REAGENT or RESOURCE	SOURCE	IDENTIFIER
**Bacterial and virus strains**		

Bacillus subtilis Z2	Laboratory Resource Library	China Center for Type Culture Collection number: CCTCC M 2020002; GenBank accession number: MT940825
Shewanella oneidensis	Laboratory Resource Library	N/A
Francisella tularensis	Laboratory Resource Library	N/A
Escherichia coli str. K-12	Laboratory Resource Library	N/A
E. coli DH5α	Thermo Scientific	CAS:EC0112

**Chemicals, peptides, and recombinant proteins**		

Cysteine	Solarbio	CAS:C0012
Ammonium sulfate ((NH4)2SO4)	Solarbio	CAS:A8821
Dipotassium hydrogenphosphate (K2HPO4)	Sangon Biotech	CAS:7758-11-4
Calcium chloride (CaCl2)	Sangon Biotech	CAS:10043-52-4
Magnesium sulfate heptahydrate (MgSO4·7H2O)	Solarbio	CAS:M8301
Ferrous sulfate (FeSO4·7H2O)	Solarbio	CAS:YZ-111613
Zinc sulfate heptahydrate (ZnSO4·7H2O)	Sangon Biotech	CAS:7446-20-0
Cobaltous chloride (CoCl2)	Sigma	CAS:7646-79-9
Lanthanum chloride (LaCl3)	Aladdin	CAS:10099-58-8
Trizol reagent	TaKaRa	Order NO. B610409
EcoR I	TaKaRa	Code NO. 1040S
Sma I	TaKaRa	Code NO. 1629
Xba I	TaKaRa	Code NO. 1634
Spe I	TaKaRa	Code NO. 1631
Hae Ⅲ	TaKaRa	Code NO. 1613

**Critical commercial assays**		

Rapid Bacterial Genomic DNA Isolation Kit	Sangon Biotech	Order NO. B518225
PrimeScript™ RT reagent Kit with gDNA Eraser	TaKaRa	Code NO. RR092S
NAD^+^/NADH Assay Kit with WST-8	Biyotime	S0175
ROS Assay Kit	Biyotime	S0033S

**Oligonucleotides**		

Primers used for gene sequences amplification, see Table S1	This paper	N/A
Primers used for spcF gene deletion, see Table S4	This paper	N/A
Primers used for quantitative RT-PCR analysis, see Table S5	This paper	N/A

### Method details

#### Strains and gene synthesis

*B. subtilis* Z2 (China Center for Type Culture Collection number: CCTCC M 2020002; GenBank accession number: MT940825) as the host for genetic transformation was screened from the composts of corn stalk in Sichuan province, China. *Shewanella oneidensis*, *Francisella tularensis* and *Escherichia coli str.* K-12 were from our laboratory. *Escherichia coli* DH5α were used for plasmid amplification. The *spcF* gene deletion mutant of *B. subtilis* Z2 (Δ*spcF*) was previously constructed and stored in our laboratory. The information and coding sequences of *nadV* gene of *S. oneidensis*, *nadM* gene of *F. tularensis*, and *sucB* gene of *E*. *coli K-12* were obtained from NCBI database, these genes were transformed into *B. subtilis* Z2 for heterologous expression using a java codon adaption tool to prevent translation from being blocked due to a shortage of tRNAs for rare codons. The restriction enzyme sites of *Bam*HI, *Eco*RI, *Sma*I, *Xba*I, *Spe*I, *Hae*Ⅲ were all avoided in the codon-optimized sequences. The optimized gene sequences were flanked by an upstream restriction enzyme prefix, an RBS site (6-10 bp, ahead of the start codon), and a downstream restriction enzyme suffix. The genome DNA was extracted using the Rapid Bacterial Genomic DNA Isolation Kit (Sangon Biotech, China) and the target genes were amplified using PrimeSTAR® Max DNA Polymerase (TaKaRa, Japan). All primers for gene sequence amplification were indicated in Table S1.

#### Bacterial culture

*B. subtilis* Z2, *S. oneidensis*, *F. tularensis*, *E. coli str.* K-12 and *E. coli* DH5α were cultured in Luria–Broth (LB) medium and were saved on LB plates at 4°C.

Prior to experiment, the seed solution of *B. subtilis* was cultivated in LB medium for 12 h at 37°C and 150 rpm. Then, bacterial cells of about 0.1 g were collected by centrifugation at 3,000×*g* and washed using 0.85% NaCl solution (w/v). Following, the collected cells were inoculated in 100 mL MBSM medium (Cysteine 0.096 g/L; (NH_4_)_2_SO_4_ 1.4 g/L; K_2_HPO_4_ 1.5 g/L; CaCl_2_ 0.1 g/L; MgSO_4_·7H_2_O 0.1 g/L; NaCl 1.0 g/L; MnSO_4_ 0.15 g/L; FeSO_4_·7H_2_O 0. 05 g/L; ZnSO_4_·7H_2_O 0.14 g/L; CoCl_2_ 0.15 g/L; pH 7.2) adding 2% (w/v) Avicel, Na 0.05 g/L and Nm 0. 05 g/L, incubated at 37°C and 150 rpm in a shaker. The samples were taken out at the inoculation of 2^nd^, 6^th^, 12^th^, 18^th^ and 24^th^ h for testing enzymatic activity and biomass concentration, or at 8^th^ h for RNA extraction and RT-qPCR analysis. The levels of protein expression, intracellular NAD(H/^+^), Ca^2+^ and ROS of wild-type (WT) strain and recombinant strains in MBSM medium were individually detected after culture of 12 h. In addition, to assess effects of the plasma membrane Ca^2+^ channels and the cytosolic Ca^2+^ levels on cellulase biosynthesis of *B. subtilis* Z2, LaCl_3_ (Aladdin, China) as an inhibitor was added into MBSM medium at a concentration of 10 mM and cultivated for 12 h.

#### Plasmid construction and transformation

All plasmid constructions were performed using pHP13-P43 as the expression vector. To the single-gene assembly in *B. subtilis*, each gene component was amplified by PCR with an upstream prefix (containing *Bam*H I), an RBS site and a downstream suffix (containing *Eco*R I). Then, these genes were individually inserted into pHP13-P43 vector to form recombinant plasmids (Figure S1A).^50^ The schematic constructions of the recombinant plasmids are shown in Figures S1B–S1D. The recombinant plasmids were verified by agarose gel electrophoresis (AGE) method (Figure S2).^51^

The above recombinant plasmids were transformed into *B. subtilis* using spizizen method.^52^ First, a single colony of *B*. *subtilis* was inoculated into 2 mL C1 medium (Table S2) and cultured for 12 h at 37°C and 220 rpm. Following, the culture solution was transferred to 4 mL fresh C1 medium for continuous incubation until the OD_600_ was up to 0.3. The cells were harvested by centrifugation at 6,000×*g* for 1 min at room temperature, and resuspended in 4 mL C2 medium again (Table S2) to cultivate for 30 min. Then, 500 μL of the culture solution were taken out and mixed with 10 μL recombinant plasmid for transformation. These recombinant cells were incubated at 37°C for 90 min. Positive transformants were selected out on LB plates containing 34 μg/mL chloramphenicol. The characteristics of all recombinant strains are shown in Table S3.

#### Construction of gene deletion mutant

For constructing a *spcF* deletion mutant, the upstream (−1 to −740 bp) and downstream (+634 to +1390 bp) fragments of *spcF* were synthesized according to the genome of *B. subtilis* Z2 using PrimeSTAR® Max DNA Polymerase (TaKaRa, Japan). All primers are indicated in Table S4. The upstream and downstream fragments of the target gene and hygromycin cassette were used as templates to perform overlap PCR. Prior to adding primers, the templates were amplified at 94°C for 3 min, 94°C for 30 S, 60°C for 30 S, 72°C for 3 min 15 S for 8 cycles. Next, the primers were added and performed 25 cycles under the same conditions. The deletion cassette of the gene was constructed by ligating the overlap PCR products to Plasmid *pEASY*-Blunt Zero Cloning Vector (TransGen Biotech, Beijing) (Figure S3A). Then, the parental strain cells were transformed by electroporation at 2.5 kV and the positive transformants were selected on the LB plate with hygromycin B and ampicillin resistance, respectively. The putative *spcF*-deleted mutants (Δ*spcF*) generated by double crossover were verified using diagnostic PCR and the primers spcF-CF1, spcF-CR1, spcF-CF2 and spcF-CR2 (Figure S3B).

#### Detections of enzymatic activity and biomass

As for FPase activity, Whatman filter paper (1 cm by 6 cm) and 0.2 mL the filter liquor was mixed in citrate buffer (pH 4.8) at 50°C for incubation of 1 h. The amount of reducing sugar released was detected using the dinitrosalicylic acid (DNS) method.^53^ The CMCase activity were measured according to the methods of Mawadza, Hatti-Kaul.^54^ 0.2 mL the filtrate of fermentation solution was mixed with 1 mL 2% (m/v) CMC-Na and incubated at 50°C for 30 min, then, amounts of the reducing sugars released were determined by the DNS method. One unit of enzyme activity was defined as the amount of enzyme required to produce 1 μmoL of reducing sugar (calculated as glucose equivalent) per minute.

As for biomass analysis, the cultured solution of 1 mL was individually collected at 2^nd^, 6^th^, 12^th^, 18^th^ and 24^th^ h after inoculation, and serially diluted with 0.85% NaCl solution, 100 μL of the diluted solution was then inoculated on LB plate and cultured at 37°C for 24 h. The bacterial colony was counted for reflecting the cell concentration in the culture solution.

#### RNA isolation and RT-qPCR analysis

The mRNA transcriptional level was assessed through RT-qPCR. The total RNA was extracted by Trizol reagent (TaKaRa, Japan), according to the manufacturer’s instructions. Synthesis of cDNA from total RNA was performed using the PrimeScript™ RT reagent Kit with gDNA Eraser (TaKaRa, Japan) as per the manufacturer’s instructions. An ABI StepOne thermocycler (Applied Biosystems, Foster City, USA) was used for qPCR. Gene transcription level was evaluated using SYBR green assays. The reaction procedure is Stage 1:Reps 1.95°C for 30 s; Stage 2:Reps 40,95°C for 5 s,60°C for 30 s 16SrRNA gene was applied to internal references to normalize the gene transcription level of target genes. The sequences of the primers for RT-qPCR are shown in Table S5. The relative gene expression levels were analyzed using the 2^−ΔΔCt^ method.

#### Measurement of protein expression levels

The protein expression levels of WT and recombinant strains were determined by sodium dodecyl sulphate polyacrylamide gel electrophoresis (SDS-PAGE) method according to Laemmli.^55^ The cells were collected by centrifugation at 8,000×*g* for 15 min at 4°C and washed thrice with 0.01 M PBS buffer (pH 7.2–7.4). The suspended cells were disrupted by sonification (HN-650Y, Shanghai, China) on ice bath, and the supernatant was harvested by centrifugation at 8,000×*g* for 20 min at 4°C for total protein analysis. Slab gels of a 4% (w/v) stacking gel and a 12% (w/v) separating gel were used to protein resolution.

#### Quantification of intracellular NAD(H/^+^) or (NAD^+^)/NADH ratio

The levels of intracellular NAD^+^ and NADH were detected by NAD^+^/NADH Assay Kit with WST-8 (Biyotime), according to the manufacturer’s instructions. The cells (about 1 g) were harvested by centrifugation at 6,000×*g* for 5 min and immediately resuspended in 200 μL ice-cooled NAD^+^/NADH extracting solution for lysing cells. The cell debris was removed by centrifugation at 12,000×*g* at 4°C for 10 min, and the supernatant was used to determine the amounts of NAD^+^ and NADH by ELIASA (imark, Bio-Rad, USA) at 450 nm.

#### Quantification of Na and Nm

The Na and Nm in the culture medium were analyzed using a high-performance liquid chromatography (HPLC) (Agilent 1260, USA). The mobile phase was KH_2_PO_4_-acetonitrile (90:10, v/v), with a flow rate of 1.0 mL min^−1^. Using a Grace Apollo C8 column (4.6 mm × 250 mm, particle size 5 μM) at an incubation temperature of 28°C.

#### Determinations of cytosolic Ca^2+^ and ROS levels

The level of cytosolic Ca^2+^ in *B. subtilis* was assessed by a Ca^2+^ fluorescent probe (Fluo-3AM). Fluo-3AM-labelled cells were detected by using a LS-55 spectrophoto fluorometer (P.E., USA) at a 488 nm excitation wavelength and 525 nm emission wavelength. The endogenous ROS level was quantified by ROS Assay Kit (Biyotime) as previously described.^56^ Ca^2+^ and ROS green fluorescence imaging analysis was performed by an inverted fluorescent microscope (DM IL LED, Leica, Germany).

### Quantification and statistical analysis

All experiments were repeated in three parallels, and the data were reported as the mean ± standard deviation (SD). Duncan’s multiple-range test was used for multiple comparisons by SPSS 19.0 (IBM Corp., NY, USA). Student’s t test was used to compare two samples by GraphPadPrism (version 8.0.2). *p* < 0.05(∗) or *p* < 0.01(∗∗) was significant.
