# Supplementary material for: Enhancing cellulase biosynthesis of Bacillus subtilis Z2 by regulating intracellular NADH level
Source: iScience. 2025 Apr 3;28(5):112341. doi: 10.1016/j.isci.2025.112341 (PMC12019202; doi:10.1016/j.isci.2025.112341)
Supplement: Document S1. Figures S1–S11 and Tables S1–S6 [file mmc1.pdf]

iScience, Volume *28*

Supplemental information

Enhancing cellulase biosynthesis of *Bacillus subtilis* Z2 by regulating intracellular NADH level

Shuai Liu, Yi Li, Lin Quan, Hai-Xia Liu, Yang Luo, and Yong-Zhong Wang

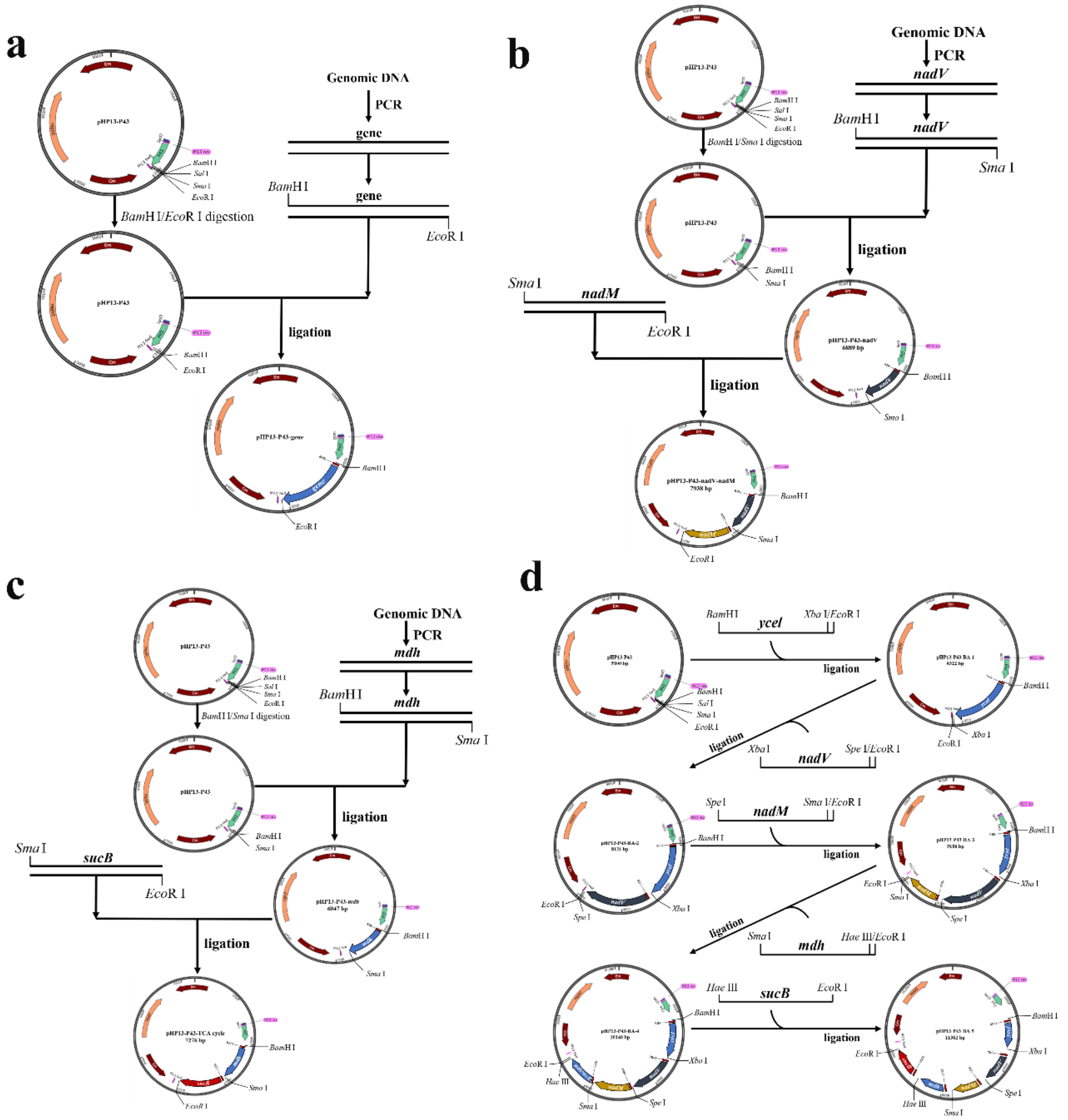

Fig. S1. The construction strategy of recombinant plasmids. The sketch maps of single-gene (a), SalvageNm-3 (b), TCA cycle-4 (c), and BA-1, BA-2, BA-3, BA-4, BA-4-1 and BA-5 (d) recombinant plasmids.

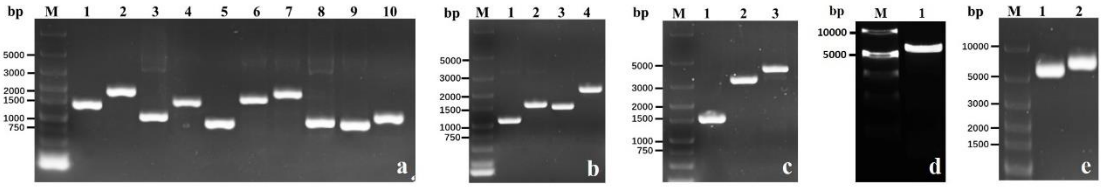

Fig. S2. The PCR verification of all recombinant plasmids. **a** A depict of PCR analysis of module 1-3. Lane 1-3 are genes *nadA*, *nadB* and *nadC*, respectively (module 1). Lane 4-7 are genes *ycel*, *pncA*, *pncB* and *nadV*, respectively (module 2). Lane 8-10 are genes *nadE*, *nadD*, *nadM* , respectively (module 3). **b** A depict of PCR analysis of module 4. Lane 1-4 are genes *mdh*, *icd*, *sucB* and assemblage of genes (*mdh* and *sucB*), respectively. **c** A depict of PCR analysis of recombinant plasmids BA-1, BA-2 and BA-3. Lane 1-3 are *ycel*, assemblage of genes (*ycel* and *nadV*) and assemblage of genes (*ycel*, *nadV* and *nadM*), respectively. **d** A depict of PCR analysis of recombinant plasmids BA-4-1. Lane 1 is assemblage of genes (*ycel*, *nadV*, *nadM* and *sucB*). **e** A depict of PCR analysis of recombinant plasmids BA-4 and BA-5. Lane 1 is assemblage of genes (*ycel*, *nadV*, *nadM* and *mdh*), lane 2 is assemblage of genes (*ycel*, *nadV*, *nadM*, *mdh* and *sucB*).

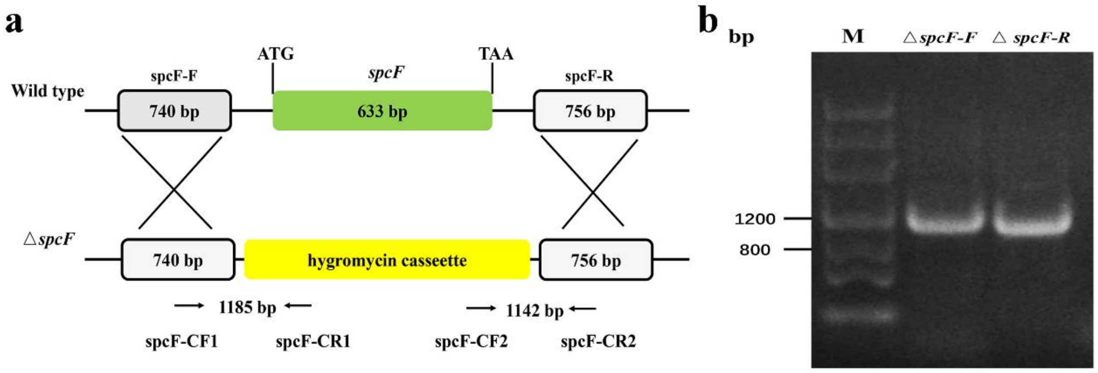

Fig. S3. Construction and verification of Δ*spcF* mutant. **a** Schematic representation of the *spcF* locus from *B. subtilis* Z2 and Δ*spcF* mutant. The binding sites of primers on the genome of *B. subtilis* Z2 and Δ*spcF*, and the expected sizes of the products in PCR verification are given. The region from +1 to +633 bp relative to the translation start site of *spcF* (green box) was replaced with the hygromycin resistance expression cassette (hygromycin, yellow box). **b** PCR verification of the Δ*spcF* mutant. Lane M, DNA molecular mass maker. PCR amplification results of the Δ*spcF*-F were obtained using spcF-CF1 with spcF-CR1 and Δ*spcF*-R were obtained using spcF-CF2 with spcF-CR2 (Table S4).

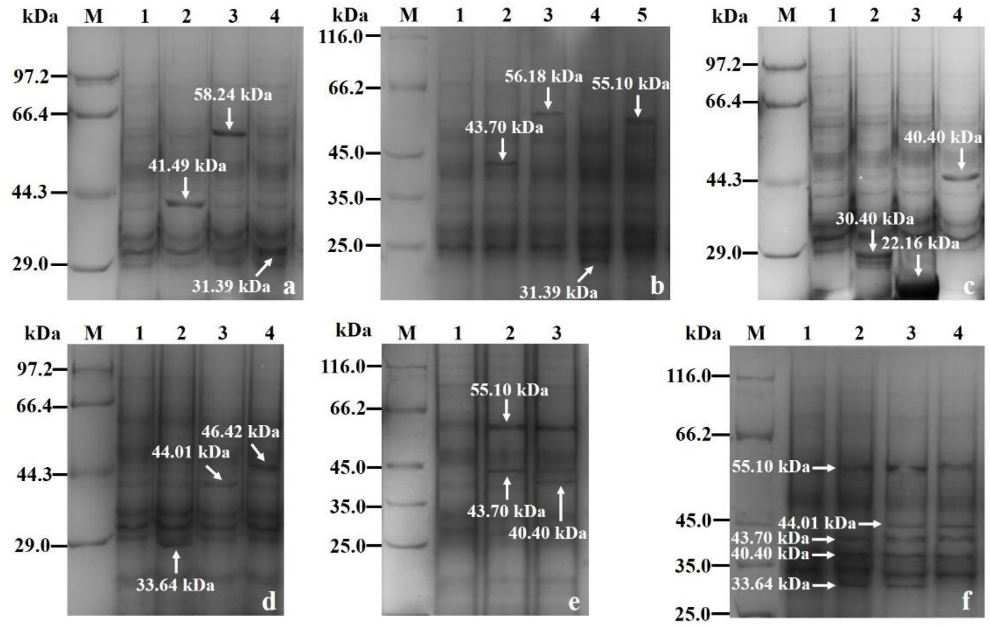

Fig. S4. The SDS-PAGE analyses of all the overexpressed genes. **a** A depict of SDS-PAGE analysis of module 1. Lane1 is the WT (control). Lane 2-4 are NadA, NadB and NadC, respectively. **b** A depict of SDS-PAGE analysis of module 2. Lane1 is the WT. Lane 2-5 are proteins niaP, PncB, PncA and NadV, respectively. **c** A depict of SDS-PAGE analysis of module 3. Lane1 is the WT. Lane 2-4 are proteins NadE, NadD and NadM, respectively. **d** A depict of SDS-PAGE analysis of module 4. Lane1 is the WT. Lane 2-4 are proteins MDH, sucB and ICDH, respectively. **e** A depict of SDS-PAGE analysis of BA-2 and BA-3. Lane1 is the WT. Lane 2 is the assemblage of proteins (niaP and NadV), lane 3 is the assemblage of proteins (niaP, NadV and NadM). **f** A depict of SDS-PAGE analysis of BA-4, BA-4-1 and BA-5. Lane1 is the WT. Lane 2 is the assemblage of proteins (niaP, NadV, NadM and MDH), lane 3 is the assemblage of proteins (niaP, NadV, NadM, MDH and sucB). lane 4 is the assemblage of proteins (niaP, NadV, NadM and sucB).

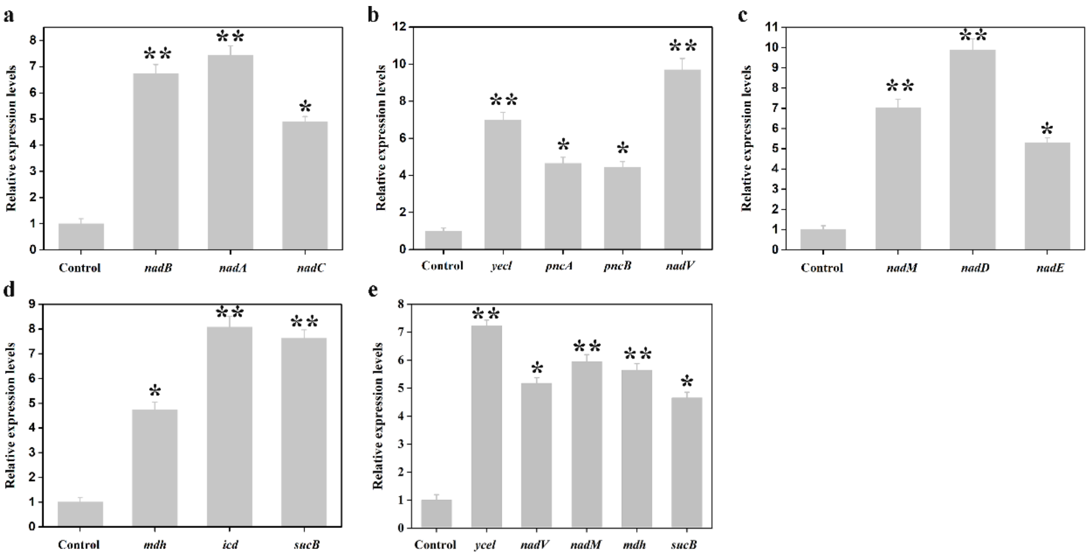

Fig. S5. The qPCR analyses of all the overexpressed genes. **a** qPCR analysis of genes *nadA*, *nadB* and *nadC* from the strans Denovo-1, Denovo-2 and Denovo-3 respectively. **b** qPCR analysis of genes *ycel*, *pncA*, *pncB* and *nadV* from the strans SalvageNa-1, SalvageNa-2, SalvageNa-3 and SalvageNm-1, respectively. **c** qPCR analysis of genes *nadM*, *nadD* and *nadE* from the strans SalvageNm-2, Universal-1 and Universal-2, respectively. **d** qPCR analysis of genes *mdh*, *icd* and *sucB* from the strans TCA cycle-1, TCA cycle-2 and TCA cycle-3, respectively. **e** qPCR analysis of genes *ycel*, *nadV*, *nadM* and *mdh* from BA-4 and BA-4-1. WT strain as a control. Values are the mean ± SD of the results from three independent experiment. Asterisks indicate significant differences (*$p < 0.05$, **$p < 0.01$, Student's t test).

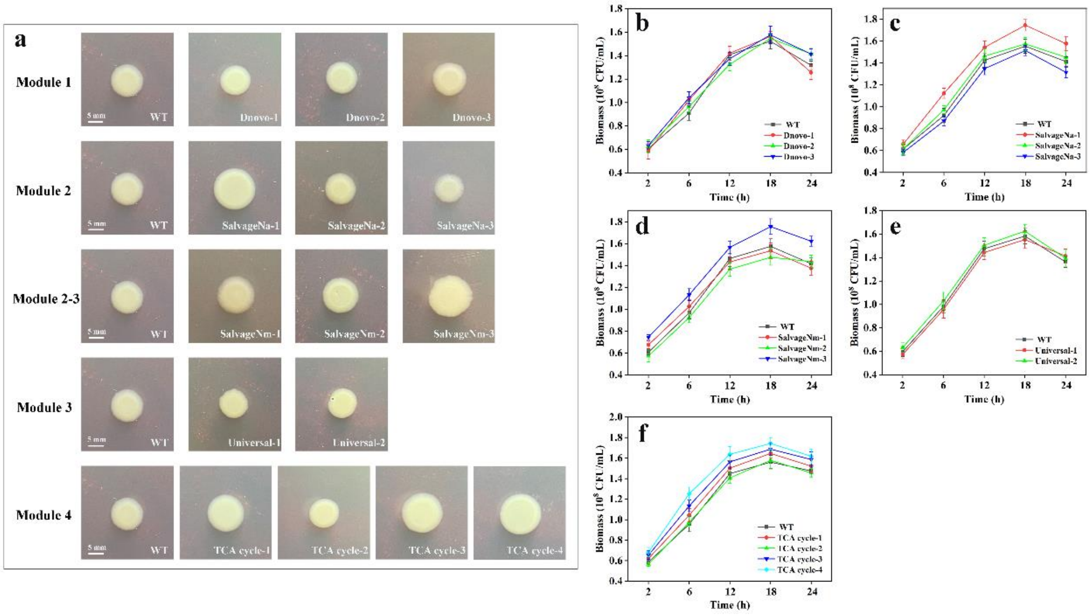

Fig. S6. The colony morphology and biomass of all recombinant strains. **a** The colony morphology of all recombinant strains Bar, 5 mm. **b** The biomass of Denovo-1, Denovo-2 and Denovo-3. **c** The biomass of SalvageNa-1, SalvageNa-2 and SalvageNa-3. **d** The biomass of SalvageNm-1, SalvageNm-2 and SalvageNm-3. **e** The biomass of Universal-1 and Universal-2. **f** The biomass of TCA cycle-1, TCA cycle-2, TCA cycle-3 and TCA cycle-4. Values are mean ± standard deviation (n = 3).

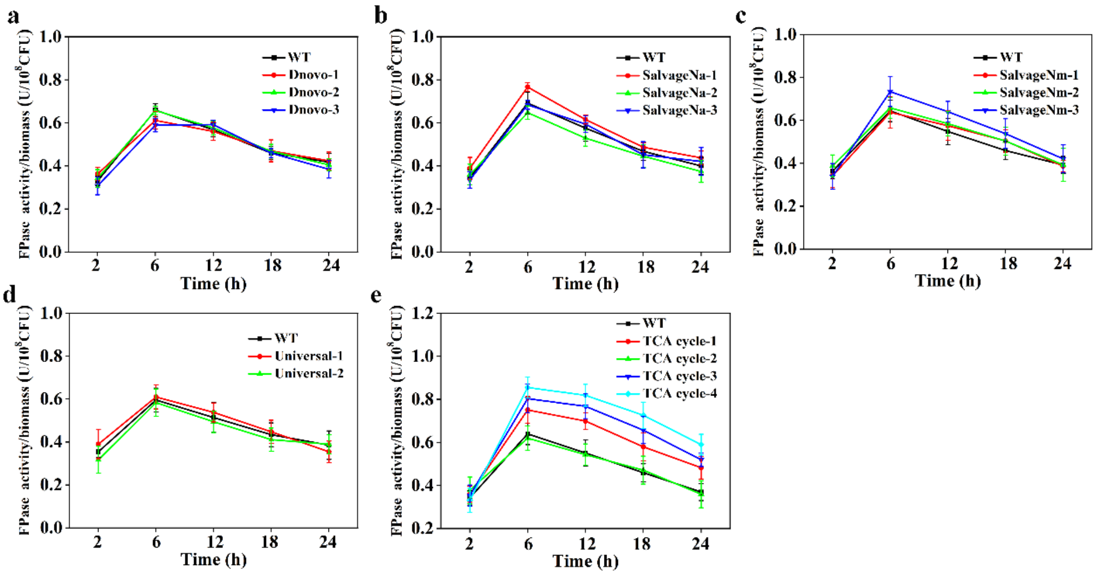

Fig. S7. The FPase activity/biomass of recombinant strains in Module 1 (**a**), in Module 2 (**b**), Module 2 and Module 3 (**c**), Module 3 (**d**) and Module 4 (**e**). The biomass was 10^8 CFU. Values are mean ± standard deviation (n = 3).

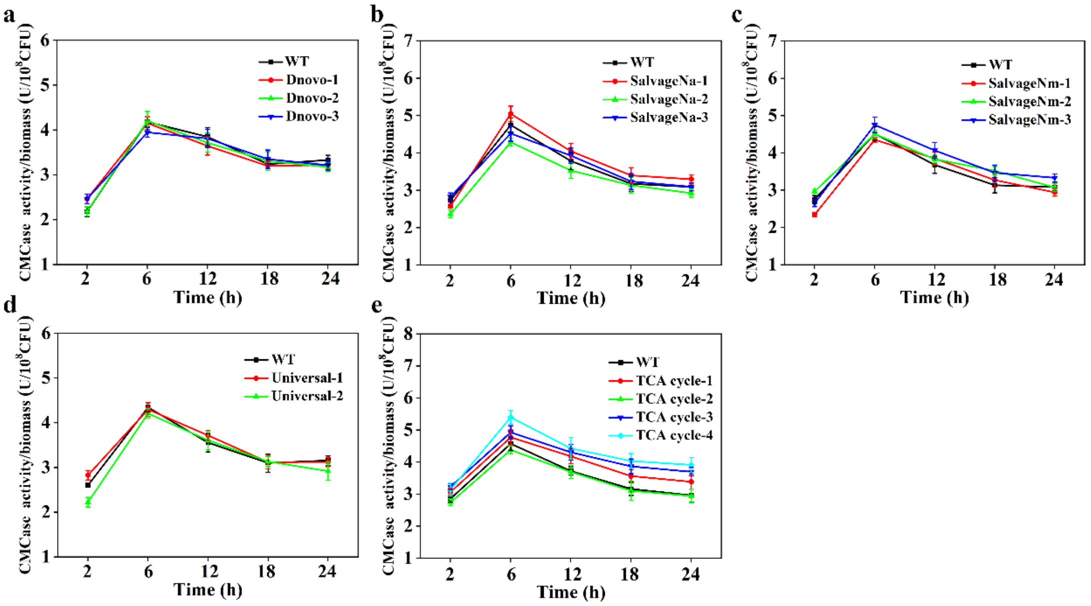

Fig. S8. The CMCase activity/biomass of recombinant strains in Module 1 (**a**), in Module 2 (**b**), Module 2 and Module 3 (**c**), Module 3 (**d**) and Module 4 (**e**). The biomass was 10^8 CFU. Values are mean ± standard deviation (n = 3).

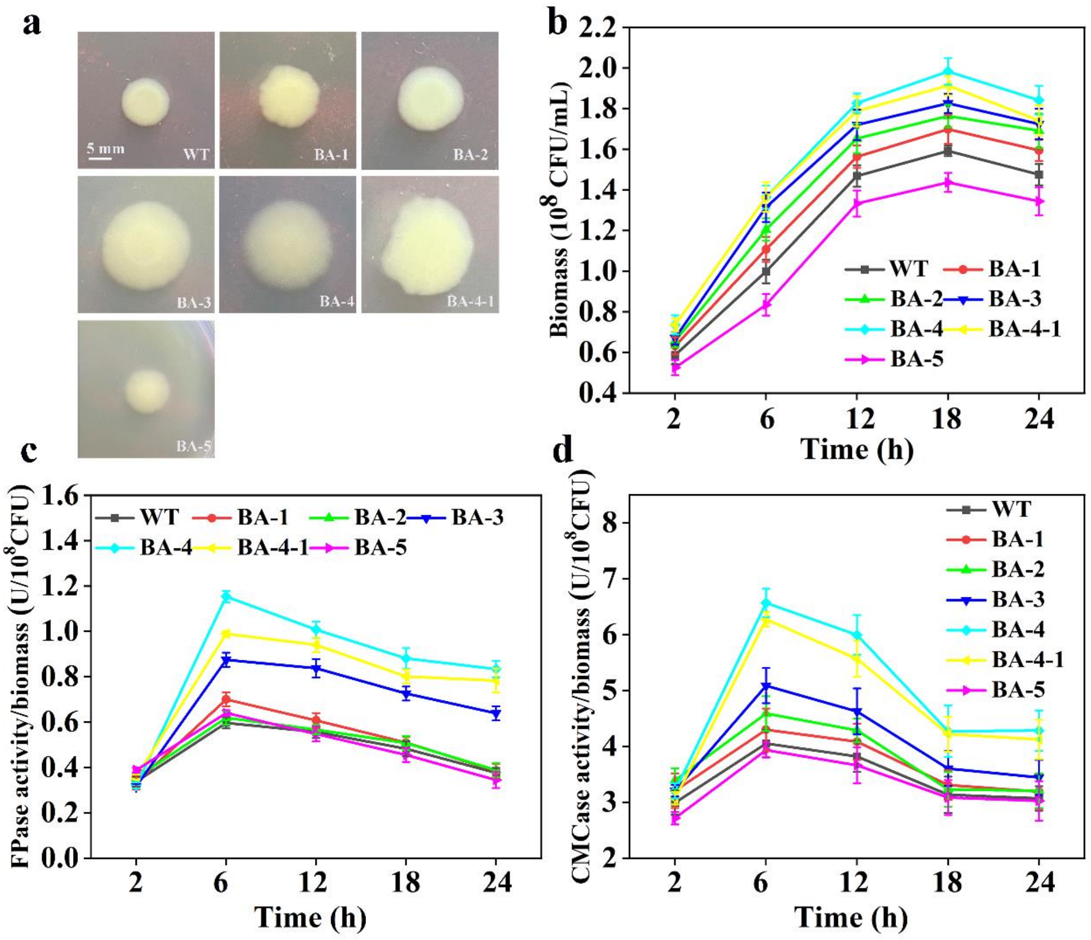

Fig. S9. The colony morphology, Bar, 5 mm (**a**), biomass (**b**), FPase activity/biomass (**c**) and CMCase activity/biomass (**d**) of recombinant strains on LB medium. The biomass was 10^8 CFU. Values are mean ± standard deviation (n = 3).

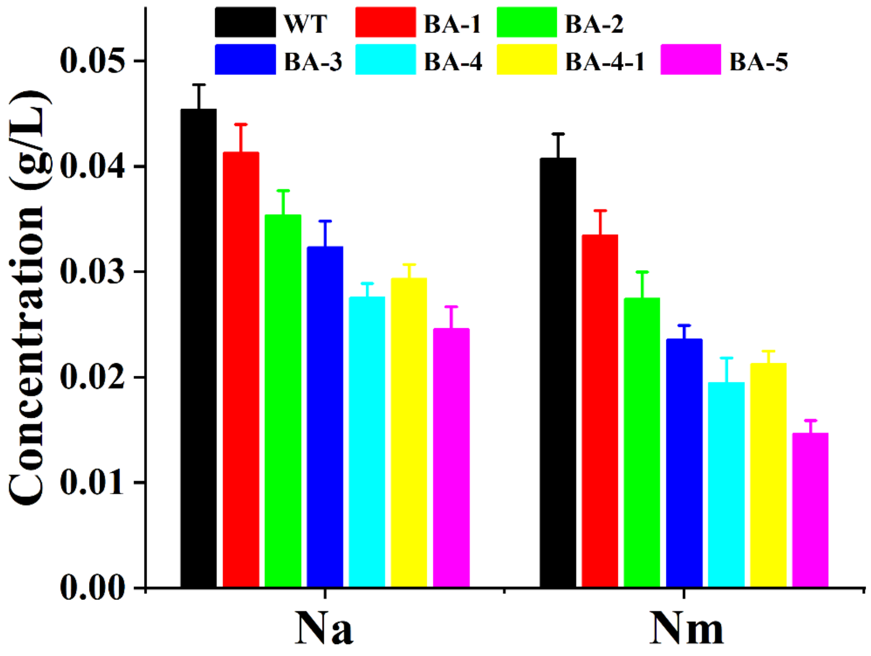

Fig. S10. Nicotinic acid (Na) and nicotinamide (Nm) concentrations in the culture medium of the recombinant *B. subtilis* Z2 strains. Values are mean ± standard deviation (n = 3).

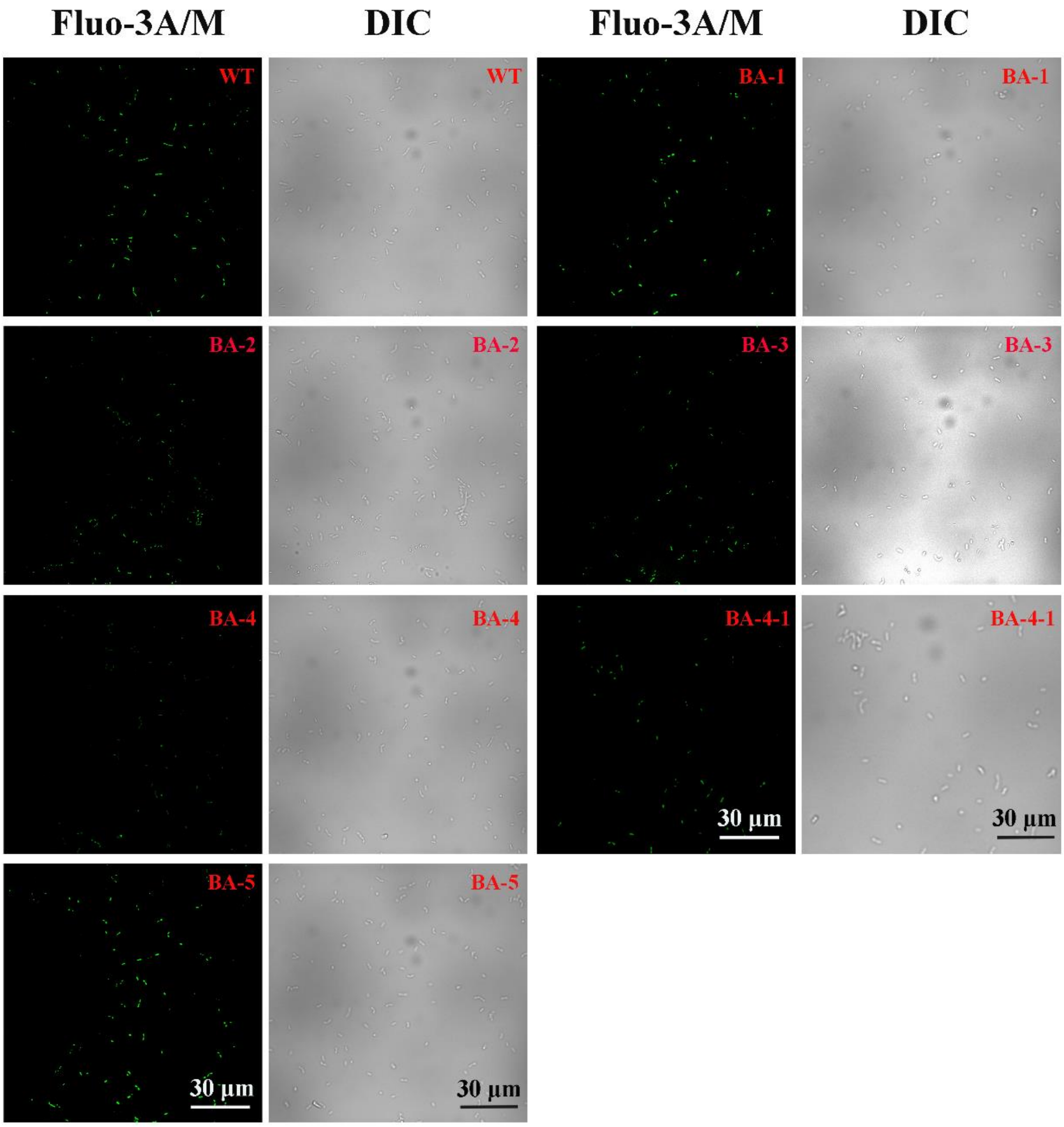

Fig. S11. Fluorescence microscopy images of ROS generation in WT, BA-1, BA-2, BA-3, BA-4, BA-4-1 and BA-5, respectively. Bar, 30 μm. Green fluorescence represents free cytosolic ROS. DIC, differential interference contrast.

Table S1. Primers used for gene sequences amplification in this study

Primer	Oligos sequences (5´to 3´)
Construction of Module 1 gene vector (Dnovo-1, Dnovo-2 and Dnovo-3)	
nadB-for	CGGGATCCCGGATGGCATATGTCTAAAA AGACGA
nadB-rev	CGGAATTCCGTCAAGAAAAAAGTGATTC
nadA-for	CGGGATCCCGAATGCTGTTATGTCAATTCTTGATG
nadA-rev	CGGAATTCCGCTACGTGATCGAAAGCA
nadC-for	CGGGATCCCGGCTGTTATGAATCATT TACAGC
nadC-rev	CGGAATTCCGTCAAGAATTGACATAA
Construction of Module 2 gene vector (SalvageNa-1, SalvageNa-2 and SalvageNa-3)	
ycel-for	CGGGATCCCGGAAATACTAGATGGGTAAACAACAACC
ycel-rev	CGGAATTCCGTTATTCTAATTCAGTTT
pncA-for	CGGGATCCCGATGCTGTTATGAAAAAAGCACTTA
pncA-rev	CGGAATTCCGTTACTCTGCCACTTGCG
pncB-for	CGGGATCCCGAAACAGTGGTGTTAGAGT ACGGATT
pncB-rev	CGGAATTCCGTTATTCTTCCTCAAGC
Construction of Module 2-3 gene vector (SalvageNm-1 and SalvageNm-2)	
nadV-for	CGGGATCCCGTGCTGTTATGACTAAATCACTTTA
nadV-rev	CGGAATTCCGTTAAAGCTGCTTGTCAAT
nadM-for	CGGGATCCCGAAAACAATGTACGATATCTCTGT
nadM-rev	CGGAATTCCGTTATAATTTTTTACCA
Construction of Module 2-3 gene vector (SalvageNm-3)	

nadV-for 1	CGGGATCCCGTGCTGTTATGACTAAATCACTTTA
nadV-rev 1	TCCCCCGGGGGATTAAAGCTGCTTGTCAAT
nadM-for 1	TCCCCCGGGGGAAACAATGTACGATATCTCTGT
nadM-rev 1	CGGAATTCCGTTATAATTTTTTACCA
Construction of Module 3 gene vector (Universal-1 and Universal-2)	
nadD-for	CGGGATCCCGAATGCTGTTATGAAGAAAATCGGAAT
nadD-rev	CGGAATTCCGTCACGATTCATATAAAC
nadE-for	CGGGATCCCGTGCTGTTATGAGCATGCAGGAAA
nadE-rev	CGGAATTCCGTTATTTCCACCAGTCA
Construction of Module 4 gene vector (TCA cycle-1, TCA cycle-2 and TCA cycle-3)	
mdh-for	CGGGATCCCGGCTGTTATGGGAAATACTCGTAA
mdh-rev	CGGAATTCCGTTAGGATAATACTTTCA
icd-for	CGGGATCCCGATGCTGGTGGCACAAGGTGAAAA
icd-rev	CGGAATTCCGTTAGTCCATGTTTTTGAT
sucB-for	CGGGATCCCGGCTGTTATGAGTAGCGTAGATA
sucB-rev	CGGAATTCCGCTACACGTCCAGCAGCAG
Construction of Module 4 gene vector (TCA cycle-4)	
mdh-for 1	CGGGATCCCGGCTGTTATGGGAAATACTCGTAA
mdh-rev 1	TCCCCCGGGGGATTAGGATAATACTTTCA
sucB-for 1	TCCCCCGGGGGAGCTGTTATGAGTAGCGTAGATA
sucB-rev 1	CGGAATTCCGCTACACGTCCAGCAGCAG
Construction of BA-1, BA-2, BA-3, BA-4, BA-4-1 and BA-5 vector	

ycel-for	CGGGATCCCGGAAATACTAGATGGGTAAACAACAACC
ycel-rev	CGGAATTCCGGCTCTAGAGC TTATTCTAATTCAGTTT
nadV-for	GCTCTAGAGCTGCTGTTATGACTAAATCACTTTA
nadV-rev	CGGAATTCCGGGACTAGTCCTTAAAGCTGCTTGTCAAT
nadM-for	GGACTAGTCCAAAACAATGTACGATATCTCTGT
nadM-rev	CGGAATTCCGTCCCCCGGGGGATTATAATTTTTTACCA
mdh-for 2	TCCCCCGGGGGAGCTGTTATGGGAAATACTCGTAA
mdh-rev 2	CGGAATTCCGGGGGCCCCTTAGGATAATACTTTCA
sucB-for 2	GGGGCCCCGCTGTTATGAGTAGCGTAGATA
sucB-rev 2	CGGAATTCCGCTACACGTCCAGCAGCAG

Table S2. Spizizen’ s minimal medium

**Mother liquor**	**C1 medium**	**C2 medium**	**Remarks**
10×Spizizen solution	600 μL	400 μL	100×Trace element solution:
Glucose solution (500 g/L)	300 μL	200 μL	$CaCl_2 \cdot 2H_2O$ 0.73 g/L,
100×Trace element solution	60 μL	40 μL	$FeCl_2 \cdot 4H_2O$ 1.35 g/L,
Casein acids Hydrolysate（20g/L）	60 μL	20 μL	$MnCl_2 \cdot 4H_2O$ 0.01 g/L, $ZnCl_2$
Tryptophan solution (5 g/L)	60 μL	4 μL	0.17 g/L, $CuCl_2 \cdot 2H_2O$ 0.043
Double distilled water	4.92 mL	3.336 mL	g/L, $CoCl_2 \cdot 6H_2O$ 0.006 g/L,
Total volume	6 mL	4 mL	$Na_2MoO_4 \cdot 2H_2O$ 0.006 g/L

Table S3. The characteristics of recombinant strains used in this study

Recombinant strains	Characteristics	References
Denovo-1	pHP13-P43-*nadB*，*B.subtilis* Z2 carries a L-aspartate oxidase gene *nadB* from *B.subtilis*, Chl ^{R}	This study
Denovo-2	pHP13-P43-*nadA*，*B.subtilis* Z2 carries a Quinolinate synthetase gene *nadA* from *B.subtilis*, Chl ^{R}	This study
Denovo-3	pHP13-P43-*nadC*，*B.subtilis* Z2 carries a Nicotinate-nucleotide pyrophosphorylase gene *nadC* from *B.subtilis*, Chl ^{R}	This study
SalvageNa-1	pHP13-P43-*ycel*，*B.subtilis* Z2 carries a Nicotinic acid and nicotinamide niaP transporter gene *ycel* from *B.subtilis*, Chl ^{R}	This study
SalvageNa-2	pHP13-P43-*pncA*，*B.subtilis* Z2 carries a Nicotinamidase gene *pncA* from *B.subtilis*, Chl ^{R}	This study
SalvageNa-3	pHP13-P43-*pncB*，*B.subtilis* Z2 carries a Nicotinate phosphoribosyl-transferase gene *pncB* from *B.subtilis*, Chl ^{R}	This study
SalvageNm-1	pHP13-P43-*nadV*，*B.subtilis* Z2 carries a Nicotinamide phosphoribosyl-transferase gene *nadV* from *S. oneidensis*, Chl ^{R}	This study
SalvageNm-2	pHP13-P43-*nadM*，*B.subtilis* Z2 carries a Nicotinamide-nucleotide adenylyltransferase gene *nadM* from *F. tularensis*, Chl ^{R}	This study
SalvageNm-3	pHP13-P43-*nadV-nadM*，*B.subtilis* Z2 carries a Nicotinamide phosphoribosyl-transferase gene *nadV* from *S. oneidensis* and a Nicotinamide-nucleotide adenylyltransferase gene *nadM* from *F. tularensis*, Chl ^{R}	This study
Universal-1	pHP13-P43-*nadD*，*B.subtilis* Z2 carries a Nicotinate-nucleotide adenylyltransferase gene *nadD* from *B.subtilis*, Chl ^{R}	This study
Universal-2	pHP13-P43-*nadE*，*B.subtilis* Z2 carries a Ammonium-dependent NAD^{+} synthetase gene *nadE* from *B.subtilis*, Chl ^{R}	This study
TCA cycle-1	pHP13-P43-*mdh*，*B.subtilis* Z2 carries a Malate dehydrogenase gene *mdh* from *B.subtilis*, Chl ^{R}	This study
TCA cycle-2	pHP13-P43-*icd*，*B.subtilis* Z2 carries a Isocitrate dehydrogenase gene *icd* from	This study

	B.subtilis, Chl ^{R}	
TCA cycle-3	pHP13-P43-*sucB*，*B.subtilis* Z2 carries a Dihydrolipoyl transsuccinylase gene *sucB* from *E. coli K-12*, Chl ^{R}	This study
TCA cycle-4	pHP13-P43-*mdh-sucB*，*B.subtilis* Z2 carries a Malate dehydrogenase gene *mdh* from *B.subtilis* and a Dihydrolipoyl transsuccinylase gene *sucB* from *E. coli K-12*, Chl ^{R}	This study
BA-1	pHP13-P43-*ycel*， *B.subtilis* Z2 carries a Nicotinic acid and nicotinamide niaP transporter gene *ycel* gene from *B.subtilis*, Chl ^{R}	This study
BA-2	pHP13-P43-*ycel-nadV*，*B.subtilis* Z2 carries a Nicotinic acid and nicotinamide niaP transporter gene *ycel* gene from *B.subtilis* and a Nicotinamide phosphoribosyl-transferase gene *nadV* from *S. oneidensis*, Chl ^{R}	This study
BA-3	pHP13-P43-*ycel-nadV-nadM*，*B.subtilis* Z2 carries a Nicotinic acid and nicotinamide niaP transporter gene *ycel* gene from *B.subtilis*, a Nicotinamide phosphoribosyl-transferase gene *nadV* from *S. oneidensis* and a Nicotinamide-nucleotide adenylyltransferase gene *nadM* from *F. tularensis*, Chl ^{R}	This study
BA-4	pHP13-P43-*ycel-nadV-nadM-mdh*，*B.subtilis* Z2 carries a Nicotinic acid and nicotinamide niaP transporter gene *ycel* gene from *B.subtilis*, a Nicotinamide phosphoribosyl-transferase gene *nadV* from *S. oneidensis*, a Nicotinamide-nucleotide adenylyltransferase gene *nadM* from *F. tularensis* and a Malate dehydrogenase gene *mdh* from *B.subtilis*, Chl ^{R}	This study
BA-4-1	pHP13-P43-*ycel-nadV-nadM- scuB*，*B.subtilis* Z2 carries a Nicotinic acid and nicotinamide niaP transporter gene *ycel* gene from *B.subtilis*, a Nicotinamide phosphoribosyl-transferase gene *nadV* from *S. oneidensis*, a Nicotinamide-nucleotide adenylyltransferase gene *nadM* from *F. tularensis* and a Malate dehydrogenase gene *mdh* from *B.subtilis*, Chl ^{R}	This study
BA-5	pHP13-P43-*ycel-nadV-nadM-mdh-scuB*，*B.subtilis* Z2 carries a Nicotinic acid and nicotinamide niaP transporter gene *ycel* gene from *B.subtilis*, a Nicotinamide phosphoribosyl-transferase gene *nadV* from *S. oneidensis*, a Nicotinamide-nucleotide adenylyltransferase gene *nadM* from *F. tularensis*, a Malate	This study

	dehydrogenase gene *mdh* from *B.subtilis* and a Dihydrolipoyl transsuccinylase gene *sucB* from *E. coli K-12*, Chl ^{R}	
Δ*spcF*::BA-3	pHP13-P43-*ycel-nadV-nadM* -Δ*spcF*，*B.subtilis* Z2 carries a Nicotinic acid and nicotinamide niaP transporter gene *ycel* gene from *B.subtilis* and a Nicotinamide phosphoribosyl-transferase gene *nadV* from *S. oneidensis*, Chl ^{R}	This study
Δ*spcF*::BA-4	pHP13-P43-*ycel-nadV-nadM-mdh-*Δ*spcF*，*B.subtilis* Z2 a Nicotinic acid and nicotinamide niaP transporter gene *ycel* gene from *B.subtilis*, a Nicotinamide phosphoribosyl-transferase gene *nadV* from *S. oneidensis*, a Nicotinamide-nucleotide adenylyltransferase gene *nadM* from *F. tularensis* and a Malate dehydrogenase gene *mdh* from *B.subtilis*, Chl ^{R}	This study

Table S4. Primers used for *spcF* gene deletion

Primer	Oligos sequences (5´to 3´)
Construction of *spcF* gene deletion vector	
spcF-FK1	CCGAGGAGCCTGACGATAAAC
spcF-RK1	TTGGTAGTTGTGACAGGACGATCCGTTTCTTCCTTTCTGT
spcF-FK2	ACAGAAAGGAAGAAACGGATCGTCCTGTCACAACTACCAA
spcF-RK2	GTCAAGAGATCCACTCCTTTCACAGATGCGTAAGGAGAAA
spcF-FK3	TTTCTCCTTACGCATCTGTGAAAGGAGTGGATCTCTTGAC
spcF-RK3	TAAAGAAAATGCAAGCGTATT
Verification of the *spcF* gene deletion mutant	
spcF-CF1	CGGTAGTTGGACTCGGGAACA
spcF-CR1	GTGCAACGCCCTTTGCAGAGC
spcF-CF2	ACTCTGATAGCTTGACTATGA
spcF-CR2	TCATGATTTCATCAAGCTGCCTT

Table S5. Primers used for quantitative RT-PCR analysis in this study

Primer	Oligos sequences (5´to 3´)
nadA-F	TTTGCGGCGTTCACT
nadA-R	ATGCCCTATTGGTCTGC
nadB-F	GGAAGGCGGATGTTTAG
nadB-R	CTCTTGGAGCCAGGTCA
nadC-F	ATGAATCATTTACAGCTGAAAAAAT
nadC-R	ATTTTTTCAGCTGTAAATGATTCAT
ycel-F	TGCGACAATGACAAGA
ycel-R	GTTTCAATTTCCACCTC
pncB-F	TCAAGTTTACGGATAGCG
pncB-R	TGCCTCCACCCTCATA
pncA-F	TATCACCCTGAAACCCG
pncA-R	GCACAACAATCCGAAAA
nadV-F	AGGTTTCCATTGGGTAG
nadV-R	TGTCGCAAGTAAGGTCA
nadE-F	AGGGAAGAACCTTGCTG
nadE-R	GGAACCTGGCGTTTATGT
nadD-F	TTGATACCGTTTCTTTACTG
nadD-R	GTGGGCTTCTTGCTTT
nadM-F	GGCAAGATGAATTACG
nadM-R	GGGAAAGAACGGATGT

mdh-F	CGGACACGGTGACGAT
mdh-R	GGTAGATGCCTTCATAGCC
sucB-F	CGCCGATCATCAACCC
sucB-R	CGTTACCAGGAAGCCCAC
icd-F	ATTTACGCAGGCATCG
icd-R	TGGCAGCTCTGACCAA
eglS-F	CATCAGCAGCAGGGAC
eglS-R	TTTCACGGACGGGTT
bglC-F	AATCCGCTTGATGGTGT
bglC-R	CCCAGTTGCTCGTTGTC
calJ-F	AAGCGGTCGGGTATG
calJ-R	CAAATGTAGAGCCAAAGG
spcF-F	ATCGCCTGCTGTTCC
spcF-R	CAATAAGACCGCCTCAA
16S-F	ACAGATTTGTGGGATTGGC
16S-R	TGTCGTGAGATGTTGGGTTA

Table S6. Comparison of different microorganisms for the cellulase production

**Microorganism**	**Substrate**	**Fermentation Conditions**	**Cellulase (CMCase) Activity**	**Reference**
Bacillus subtilis K-18	Potato peel	50 °C, 24 h	3.50 ± 0.11 U/mL	1
Bacillus subtilis BS-5	lactose	35 °C, 48 h	9.6 U/ml	2
*Bacillus subtil*is Z2	kali-pretreated rice straw	37 °C, 12 h	6.74 ± 0.14 U/mL	3
Bacillus Subtilis VS15	cotton ginning waste	37 °C, 54 h	10.02 ± 0.03 U/mL	4
Streptomyces fulvissimus CKS7	Rye bran	30 °C, 6 days	8.62 ± 0.08 U/mL	5
Hymenobacter sp. CKS3	5.0% corn stover, 2.5% molasses	4 days	1.11 U/mL	6
Aspergillus niger ITV 02	Delignified sweet sorghum bagasse	30 °C, 50 h	0.61 ± 0.025 U/mL	7
Bacillus subtilis Z2	Avicel	37 °C, 12 h	10.95 ± 1.78 U/mL	This study

References

1. Irfan, M., Mushtaq, Q., Tabssum, F., Shakir, H.A., Qazi, J.I. (2017). Carboxymethyl cellulase production optimization from newly isolated thermophilic *Bacillus subtilis* K-18 for saccharification using response surface methodology. AMB Express 7: 29. https://doi.org/ 10.1186/s13568-017-0331-3.

2. Xu, J., Gao, Z., Wu B., He, B.F. (2017). Lactose-inducted production of a complete lignocellulolytic enzyme system by a novel bacterium *Bacillus* sp. BS-5 and its application for saccharification of alkali-pretreated corn cob. Cellulose 24:2059-2070. https://doi.org/ 10.1007/s10570-017-1247-4.

3. Liu, S., Gao, Y.W., Quan, L., Yang, M., Wang, Y.Z., Hou, C.J. (2022). Improvement of lignocellulolytic enzyme production mediated by calcium

signaling in *Bacillus subtilis* Z2 under graphene oxide stress. Appl. Environ. Microbiol. 88(19). https://doi.org/ 10.1128/aem.00960-22.

4. Ega, S.L., Drendel, G., Petrovski, S., Egidi, E., Franks, A.E., Muddada, S. (2020). Comparative analysis of structural variations due to genome shuffling of *Bacillus subtilis* vs15 for improved cellulase production. Int. J. Mol. Sci. 21(4). https://doi.org/ 10.3390/ijms21041299.

5. Mihajlovski, K., Bunti´c, A., Mili´c, M., Rajili´c-Stojanovi´c, M., Dimitrijevi´c-Brankovi´c, S. (2021). From agricultural waste to biofuel: Enzymatic potential of a bacterial isolate *Streptomyces fulvissimus* CKS7 for bioethanol production. Waste Biomass Valorization 12:165-174. https://doi.org/ 10.1007/s12649-020-00960-3.

6. Mihajlovski, K., Pecarski, D., Rajili´c-Stojanovi´c, M., Dimitrijevi´c-Brankovi´ c, S. (2021). Valorization of corn stover and molasses for enzyme synthesis, lignocellulosic hydrolysis and bioethanol production by *Hymenobacter* sp. CKS3. Environ. Technol. Innov. 23:101627. https://doi.org/ 10.1016/j.eti.2021.101627.

7. Infanzón-Rodríguez, M., Ragazzo-Sánchez, J., Del Moral, S., Calderón-Santoyo, M., Gutiérrez-Rivera, B., Aguilar-Uscanga, M. (2020). Optimization of cellulase production by *Aspergillus niger* ITV 02 from sweet sorghum bagasse in submerged culture using a Box–Behnken design. Sugar Tech. 22: 266-273. https://doi.org/ 10.1007/s12355-019-00765-2.
